# Transcriptome Comparison of Defense Responses in the Rice Variety ‘Jao Hom Nin’ Regarding Two Blast Resistant Genes, *Pish* and *Pik*

**DOI:** 10.3390/plants9060694

**Published:** 2020-05-29

**Authors:** Athipat Ngernmuen, Worrawit Suktrakul, Sureeporn Kate-Ngam, Chatchawan Jantasuriyarat

**Affiliations:** 1 Department of Genetics, Faculty of Science, Kasetsart University, Bangkhen Campus, Ladyao, Chatuchak, Bangkok 10900, Thailand; athipat.ngern@gmail.com (A.N.); worrawit.s@ku.th (W.S.); 2 Department of Agronomy, Faculty of Agriculture, Ubon Ratchathani University, Warinchamrap, Ubon Ratchathani 34190, Thailand; sureeporn.k@ubu.ac.th; 3 Center for Advanced Studies in Tropical Natural Resources, National Research University-Kasetsart University (CASTNAR, NRU-KU), Kasetsart University, Bangkok 10900, Thailand; 4 Omics Center for Agriculture, Bioresources, Food and Health, Kasetsart University (OmiKU), Kasetsart University, Bangkok 10900, Thailand

**Keywords:** DNA marker, graphical genotype, rice blast disease, RNA-seq, transcriptome

## Abstract

Jao Hom Nin (JHN) is a Thai rice variety with broad-spectrum resistant against rice blast fungus. JHN contains two rice blast resistant genes, *Pish* and *Pik*, located on chromosome 1 and on chromosome 11, respectively. To understand the blast resistance in JHN, the study of the defense mechanism related to the *Pish* and *Pik* genes is crucial. This study aimed to dissect defense response genes between the *Pish* and *Pik* genes using the RNA-seq technique. Differentially expressed genes (DEGs) of *Pish* and *Pik* backcross inbred lines were identified between 0 and 24 h after inoculation with rice blast spore suspension. The results showed that 1248 and 858 DEGs were unique to the *Pish* and *Pik* lines, respectively. The wall-associated kinase gene was unique to the *Pish* line and the zinc-finger-containing protein gene was unique to the *Pik* line. Pathogenicity-related proteins PR-4 and PR-10 were commonly found in both *Pish* and *Pik* lines. Moreover, DEGs functionally categorized in brassinosteriod, jasmonic acid, and salicylic acid pathways were detected in both *Pish* and *Pik* lines. These unique and shared genes in the *Pish* and *Pik* rice blast defense responses will help to dissect the mechanisms of plant defense and facilitate rice blast breeding programs.

## 1. Introduction

Rice, *Oryza sativa* L., is an important cereal crop and serves as a major source of energy for more than half of the global population. In the past decade, the total global rice production each year was more than 700 million tons, which was mainly produced in Asian countries, such as Thailand, India, Vietnam, and China [1,2]. Currently, a challenge in rice production is the declining rice productivity due to both abiotic and biotic stresses, which include insect pests, bacteria, and fungi [3]. Rice blast disease, caused by an ascomycete fungus *Magnaporthe oryzae*, is one of the most destructive diseases in rice production worldwide. All above-ground rice plants can be infected by this fungus. This disease frequently occurs in temperate rice growing regions. An outbreak of the rice blast disease results in rice yield loss. The rice blast disease is responsible for 30% global rice production losses and results in an increase of costs to control this disease [4,5]. One of the potential methods to control this disease is using rice-blast-resistant cultivars [6]. The resistance capability of the resistant cultivars is explained by a gene-for-gene interaction, which is an interaction between resistance proteins (R proteins) in plants and avirulence proteins (Avr proteins) in pathogens. The resistant cultivars contain R proteins that correspond with Avr proteins, which trigger a plant defense response, leading to plant resistance [7,8]. Plant defense against pathogen initiates with pathogen recognition. After this, a signal transduction is mediated by several actions, including a phosphorylation–dephosphorylation cascade. The outcomes of plant defense response are a hypersensitive response (HR), a programmed cell death, and an expression of defense-related genes to inhibit the pathogen growth [9,10]. To date, there are more than 100 *R* genes mapped in the rice genome [11]. Some cloned genes have been molecularly characterized. Most cloned blast *R* genes encode proteins, encoding a nucleotide binding site (NBS) and leucine-rich repeats (LRR) [12,13]. For example, the *Pish* and *Pik* genes have been cloned and characterized as the NBS-LRR genes [14,15]. The *Pish* gene shows partial resistance to rice blast isolates, allowing some pathogen growth [14,16]. On the other hand, the *Pik* gene shows complete resistance to rice blast isolates [15,16]. The improvement of the resistant rice cultivars by an introgression of the desirable resistance genes into susceptible cultivars provides the best way to protect rice from this disease. Marker-assisted selection (MAS), the breeding procedure that integrates the DNA marker detection and selection into a traditional breeding program, is used to produce the resistant rice cultivars [17,18]. Graphical genotype, the display of the proportion of donor and recurrent genomes in the subsequent backcross generations, allows the selection of the desirable lines with the resistance-related genes and resistant phenotypes [19]. These selected lines can be used to investigate the gene expression that reflects how the resistant cultivars were able to overcome the pathogen.

A transcriptome is a whole set of transcribed RNAs from a specific tissue or cell type at a developmental stage or under certain conditions [20]. Several transcriptomic studies related to the rice blast resistance using various rice varieties and blast isolates have been previously reported [21,22,23,24,25]. The RNA-seq was used to dissect the molecular defense mechanism in the early response at 24 h after blast inoculation in Gigante Vercelli (a durable and broad resistant rice variety) and Vialone Nano (a highly susceptible rice variety) [26]. The resistant cultivar showed upregulation of defense-response-related genes encoding diterpene phytoalexin biosynthetic enzymes, flavin-containing monooxygenase, class I chitinase, and glycosyl hydrolase17. The genes related to the early steps of defense perception signaling, including chitin oligosaccharides sensing factors, wall associated kinases, MAPK cascades, and WRKY transcription factors, were also detected [26].

The Thai rice variety named Jao Hom Nin (JHN) is a non-glutinous rice variety with resistance to leaf and neck blast diseases under natural conditions [27]. JHN demonstrates a broad-spectrum resistance against various rice blast isolates in Thailand. A previous report showed that JHN contains two rice blast resistance quantitative trait loci (QTLs), located on chromosome 1 and on chromosome 11 [28]. These two QTLs were introgressed into a Thai glutinous rice cultivar RD6, a rice-blast-susceptible cultivar [29]. Later, the two resistance genes in JHN were cloned and revealed to be the *Pish* gene on chromosome 1 and the *Pik* gene on chromosome 11 [30]. In this study, we obtained nine backcross inbred lines from a cross between RD6 (the recurrent parent) and JHN (the donor parent). These inbred lines were classified into three groups: three lines containing QTL on chromosome 1 of JHN (labelled as qBL1), three lines containing QTL on chromosome 11 of JHN (qBL11), and three lines containing both QTLs on chromosome 1 and 11 (qBL1 and 11). Polymorphic DNA markers between RD6 and JHN were used to construct a graphical genotype to examine the genetic makeup of all nine inbred lines in order to select the representative lines for the transcriptomic analysis. RNA-seq technique was used to identify the defense response transcripts in rice containing the *Pish* and *Pik* genes. The data generated from this transcriptomic study revealed the common and unique defense response pathways of the two different rice blast resistance genes and will lead to understanding the mechanism of rice blast resistance in the JHN rice cultivar.

## 2. Results

### 2.1. Graphical Genotyping of RD6 Backcross Inbred Lines

A total of 363 selected rice DNA markers consisting of 133 InDel markers (Table S1) obtained from a previous report of rice InDel marker development [31] and 230 SSR markers (Table S2) from GRAMENE database were used to determine the polymorphism between JHN rice cultivar (resistant variety) and RD6 (susceptible variety). In total, 94 out of 363 markers (24.7%) showed polymorphism between JHN and RD6, including 18 InDel markers and 76 SSR markers. The number of the polymorphic markers between JHN and RD6 in each chromosome ranged from 5 to 13 markers. Chromosomes 1, 2, 3, 4, 6, 9, 10, 11, and 12 contain both InDel and SSR markers, but chromosomes 5, 7, and 8 only contain SSR markers (Table S3). These 94 polymorphic markers were consequently used for the graphical genotype construction of nine backcross inbred lines (BILs).

**Figure 1:**
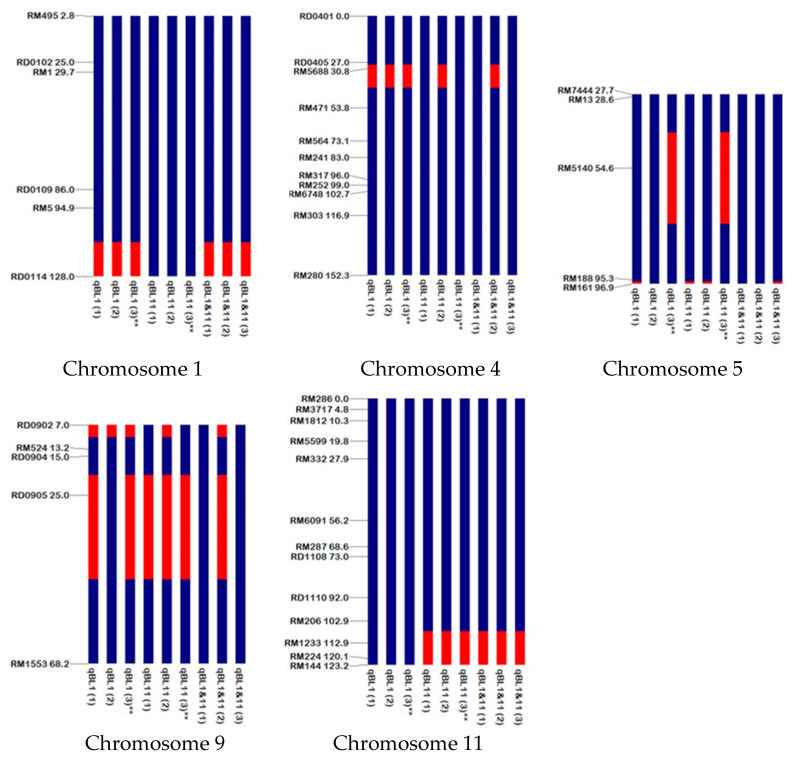
Graphical genotype of nine backcross inbred lines (BILs) showing the genetic backgrounds of Jao Hom Nin (JHN) (red) and RD6 (blue), which were constructed by GGT 2.0: Graphical GenoTyping. Note: line names ending with double asterisk indicate the selected lines in the subsequent transcriptome analysis.

The graphical genotype of nine backcross inbred lines was constructed by GGT 2.0: Graphical GenoTyping. The graphical genotyping-illustrated RD6 genetic background ranged from 92.55 to 96.81 percent (Table S4). Chromosomes 2, 3, 6, 7, 8, 10, and 12 had the entire RD6 genetic background, while chromosomes 1, 4, 5, 9, and 11 had the introgression fragments from JHN (Figure 1). To select the representatives of BILs for subsequent transcriptomic study, BILs with the highest percentage of RD6 background with a sufficient amount of seeds were selected, including the qBL1 (3) line, representing the BIL line containing the *Pish* gene; and the qBL11 (3) line, representing the BIL line containing the *Pik* gene. These lines were designated as Q1 and Q11, respectively, in transcriptomic analysis.

### 2.2. Transcriptome Sequencing Data of Rice Containing Pish and Pik Genes upon Rice Blast Infection

**Figure 2:**
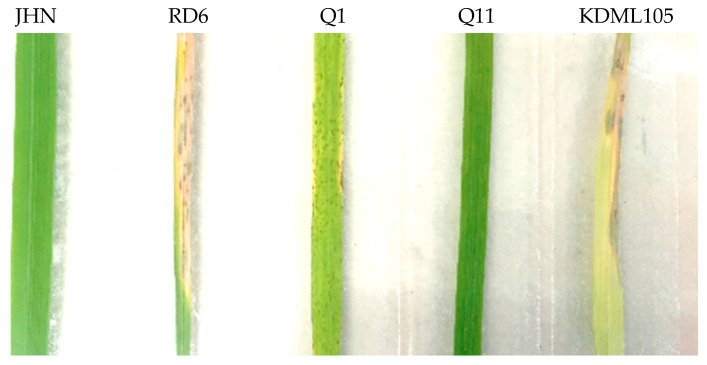
Pathogenicity of rice varieties and BIL lines inoculated with rice blast isolate THL84. Q1 and Q11 are BIL containing the *Pish* and *Pik* genes in the RD6 background, respectively.

Two replications of 21-day-old seeding of JHN, RD6, Q1 (the selected rice line containing the *Pish* gene), and Q11 (the selected rice containing the *Pik* gene) were inoculated with rice blast isolate THL84. The leaf samples were collected at 0 and 24 h after inoculation. After 7 days of inoculation, the disease reaction was observed. RD6 and Q1 plants showed rice blast disease symptoms, while JHN and Q11 were resistant to rice blast isolate THL84. KDML105, a Thai rice cultivar, was used as the susceptible control (Figure 2). RNA was extracted from leaf samples and used for the RNA-seq experiment. The RNA-seq results showed that each sample had total reads ranging between 11,724,286 and 15,418,460 reads, while the total mapped reads ranged between 92.7% and 93.5%. The reads with multiple alignments represented approximately 6% of the total (Table S5). The data have been deposited with links to BioProject accession number PRJNA634330 in the DDBJ BioProject database.

### 2.3. Identification of Differently Expressed Genes (DEGs) between 0 and 24 H After Innoculation in the Pish-Containing Rice (Q1) and the Pik-Containing Rice (Q11)

**Figure 3:**
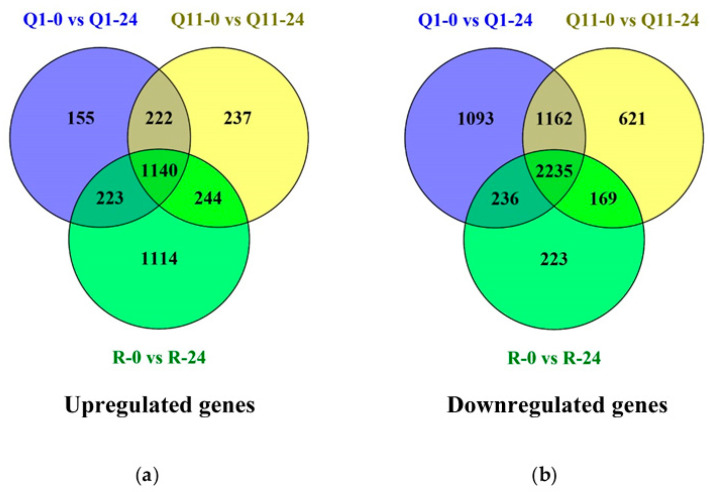
Venn diagram showing numbers of up- or downregulated genes in RD6, Q1, and Q11 rice lines at 0 and 24 h after inoculation with rice blast fungus isolate THL84: (**a**) upregulated genes and (**b**) downregulated genes. This Venn diagram was constructed by Venny version 2.1.0.

Leaf samples of the *Pish*-containing rice line Q1 collected at 0 and 24 h after inoculation (hai) were named Q1-0 and Q1-24, respectively; and leaf samples of the *Pik*-containing rice line Q11 collected at 0 and 24 hai were named Q11-0 and Q11-24, respectively. Moreover, leaf samples of the RD6 rice variety, the negative control, collected at 0 and 24 hai were named R-0 and R-24, respectively. Cuffdiff was used to determine the number of differentially expressed genes (DEGs). There were 5584 DEGs, including 2721 upregulated genes and 2863 downregulated genes. There were 155 upregulated and 1093 downregulated DEGs unique to the *Pish* gene that were only presented in Q1. There were 237 upregulated and 631 downregulated DEGs unique to the *Pik* gene that were only presented in Q11. There were 222 upregulated and 1162 downregulated DEGs that were commonly found in both Q1 and Q11 (Figure 3). Principal component analysis (PCA) showed that gene expression profiles of the samples at 0 hai were clustered together, while those of the samples at 24 hai were scattered. Moreover, the results indicated that the expression profiles between 0 and 24 hai were highly different, suggesting the different responses of RD6, Q1, and Q11 lines to rice blast infection (Figure S4).

The top 20 upregulated DEGs unique to Q1 or Q11 are listed in Table 1, while those DEGs that were common between Q1 and Q11 are listed in Table 2. Some of these genes were hypothetical genes and unknown function proteins. The genes encoding the late embryogenesis abundant (LEA) protein, germin-like protein, pathogenesis-related transcriptional factor, wall-associated kinase, metallothionein-like protein type 1, dehydrin RAB 16D, lipase, GA 2-oxidase3, auxin influx carrier protein, and ATCNGC15 protein were unique to Q1. Additionally, the genes encoding guanine nucleotide exchange factors for Rop, zinc-finger-containing protein, atypical basic helix–loop–helix protein, similar P-type R2R3 Myb protein, MADS box transcription factor, alpha/beta hydrolase fold-3 domain, chitinase III C10150, GAST, bifunctional inhibitor protein, esterase, and lipase were unique to Q11 (Table 2). 

Moreover, 27 upregulated DEGs between R-0 and Q1-0 were identified. These genes encode for Myb/SANT-like domain containing protein, a beta subunit of polygalacturonase 1 and peroxidase. Another 32 upregulated DEGs between R-0 and Q11-0 were also identified. These genes encode for isoflavone reductase homolog IRL, mitochondrial chaperonin-60, and cytosolic pyruvate orthophosphate dikinase (Table S6).

### 2.4. Identification of Gene Ontology Using agriGO

**Figure 4:**
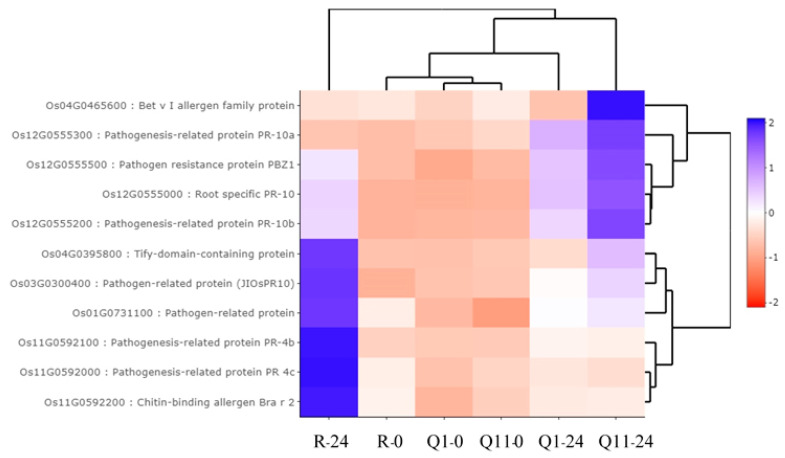
Heat map analysis, indicating expression patterns of 11 DEGs in the defense response group. Note: R-0, Q1-0, and Q11-0 indicate RD6, Q1, and Q11 lines at 0 h after inoculation; and R-24, Q1-24, and Q11-24 indicate RD6, Q1, and Q11 lines at 24 h after inoculation.

To examine the function of differentially expressed genes in the *Pish*-containing rice line (Q1-0 vs. Q1-24), the *Pik*-containing rice line (Q11-0 vs. Q11-24), and RD6 rice variety (R-0 vs. R-24), the gene ontologies were classified by GO terms into three groups based on their biological process, molecular function, and cellular components, using GO Analysis toolkit and database for agricultural communities, or agriGO v2.0. The major GO term in terms of biological function was the carbohydrate metabolic process (GO:0005975). The major GO term in terms of molecular function was the protein tyrosine kinase activity (GO:0004713). The major GO term in terms of the cellular component was the part (GO:0044464) (Figures S1 and S2). Interestingly, several DEGs were classified in the defense response group, including biotic stimulus (GO:0009607), defense response (GO:0006952), and defense response to fungus (GO:0050832). The examples of these DEGs were pathogenesis-related protein PR-10a, pathogenesis-related protein PR-4b, Tify-domain-containing protein, root specific PR-10, pathogen resistance protein PBZ1, and the pathogenesis-related protein PR-10b (Figure 4).

### 2.5. Identification of Biological Pathway Using Plant Reactome

The differentially expressed genes were categorized based on their function by Plant Reactome (Tables S7 and S8). The categorized functions in Plant Reactome database were hormone, biosynthesis, signaling and transport. The results showed that some differentially expressed genes were related to brassinosteriods, jasmonic acid, and salicylic acid. 

**Figure 5:**
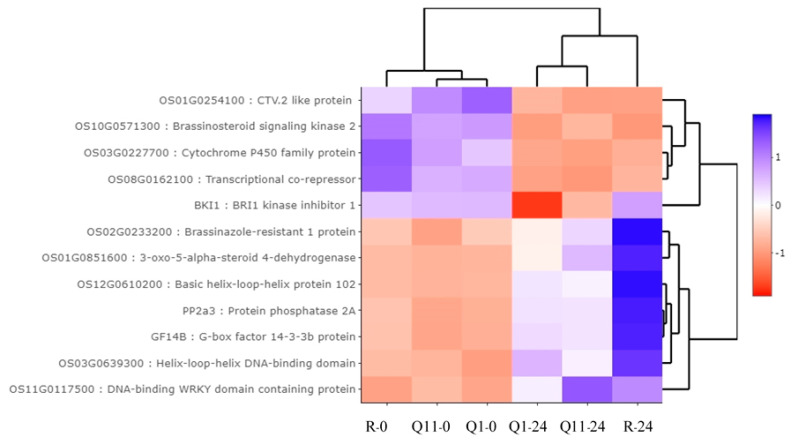
Heat map analysis indicating expression pattern of 12 differentially expressed genes in brassinosteriod-related genes by Plant Reactome database. Note: R-0, Q1-0, and Q11-0 indicate RD6, Q1, and Q11 lines at 0 h after inoculation; and R-24, Q1-24, and Q11-24 indicate RD6, Q1, and Q11 lines at 24 h after inoculation.

Twelve DEGs were classified into the brassinosteriod related group, which includes the genes in brassinosteriod biosynthesis and genes in brassinosteriod signaling. Based on their expression pattern, seven genes were upregulated at 24 hai and five genes were downregulated at 24 hai. Seven upregulated genes were 3-oxo-5-alpha-steroid 4-dehydrogenase, G-box factor 14-3-3b protein, brassinazole-resistant 1 protein, helix–loop–helix DNA-binding domain, DNA-binding WRKY domain containing protein and basic helix–loop–helix protein 102. Five downregulated genes were BRI1 kinase inhibitor 1, CTV.2-like protein, cytochrome P450 family protein, transcriptional co-repressor, brassinosteriod signaling kinase 2, and protein phosphatase 2A (Figure 5).

**Figure 6:**
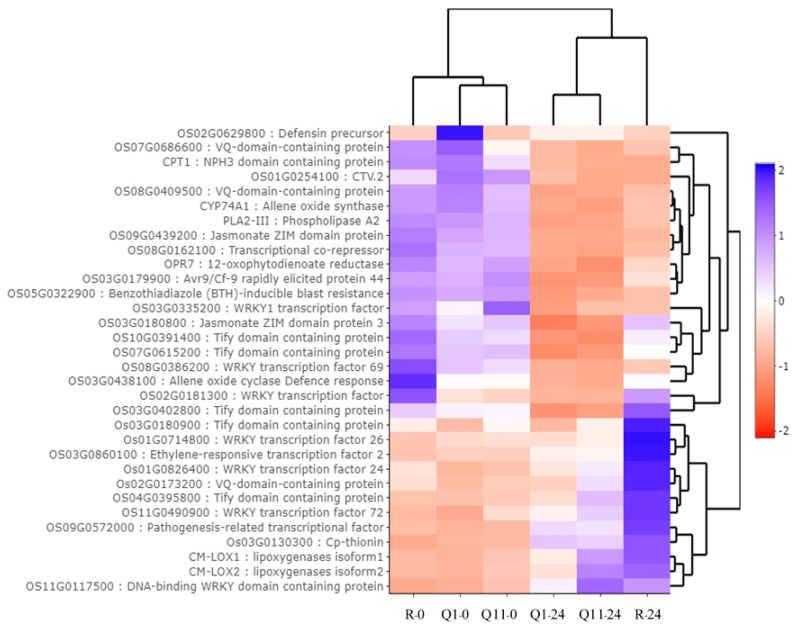
Heat map analysis indicating expression pattern of 32 differently expressed genes in jasmonic-acid-related genes by Plant Reactome database. Note: R-0, Q1-0, and Q11-0 indicate RD6, Q1, and Q11 lines at 0 h after inoculation; and R-24, Q1-24, and Q11-24 indicate RD6, Q1, and Q11 lines at 24 h after inoculation.

Thirty-two DEGs were classified into the jasmonic-acid-related gene group. These genes were involved in a jasmonic acid biosynthesis and in a jasmonic acid signaling. Based on their expression pattern, thirteen genes were upregulated at 24 hai and 19 genes were downregulated at 24 hai. These upregulated genes encode for lipoxygenases isoform 1, lipoxygenases isoform 2, ethylene-responsive transcription factor 2, Tify domain containing proteins, pathogenesis-related transcriptional factor, DNA-binding WRKY domain containing protein, WRKY transcription factor 72, WRKY transcription factor 26, WRKY transcription factor 24, VQ-domain-containing protein, and Cp-thionin. The downregulated genes encode for allene oxide synthase, 12-oxophytodienoate reductase, VQ-domain-containing protein, and jasmonate ZIM domain protein (Figure 6).

**Figure 7:**
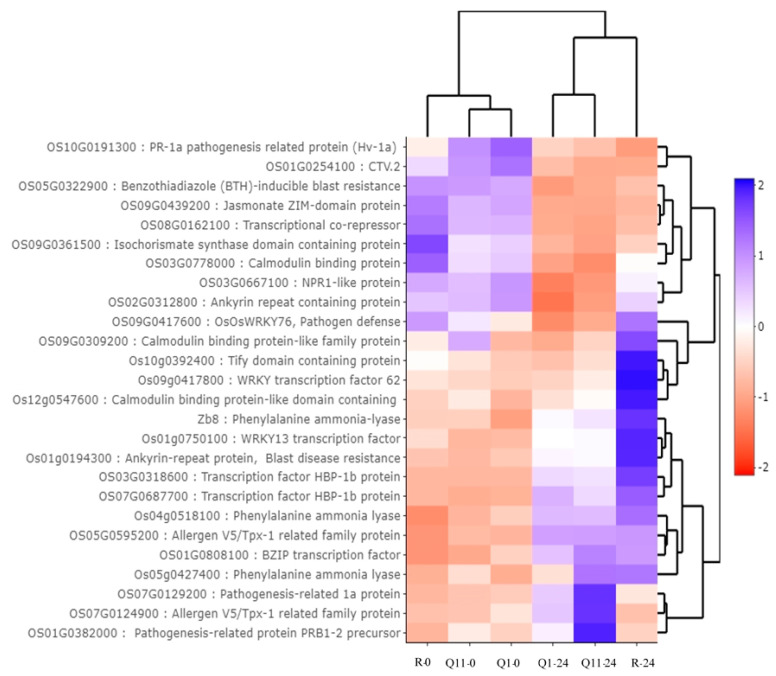
Heat map analysis indicating expression patterns of 26 differentially expressed genes in salicylic acid signaling by Plant Reactome database. Note: R-0, Q1-0, and Q11-0 indicate RD6, Q1, and Q11 lines at 0 h after inoculation; and R-24, Q1-24, and Q11-24 indicate RD6, Q1, and Q11 lines at 24 h after inoculation.

Twenty-six DEGs were classified in salicylic acid signaling. Based on their expression patterns, nine genes were upregulated at 24 hai. These genes encode for transcription factor HBP-1b proteins, allergen V5/Tpx-1 related family proteins, WRKY transcription factor 62, calmodulin binding protein, phenylalanine ammonia lyases, and WRKY13 transcription factor (Figure 7).

### 2.6. Validation of RNA-Seq Results by a Quantitative Real-Time PCR (qRT-PCR)

To validate the RNA-seq gene expression results, ten DEGs with log_2_FC values higher than 3 were chosen for a quantitative real-time PCR analysis. The three upregulated genes that were common among Q1, Q11, and RD6 (Os10G0549000, Os07G0529000, and Os08G01576000), two upregulated that were common between Q1 and Q11 (Os09G0319800 and Os11G0701500), two upregulated genes that were unique to Q1 (Os06G0573500 and Os10G0180800), and three upregulated genes that were unique to Q11 (Os02G0514150, Os11G0701400, and Os11G0134950) were examined. The comparison between the RNA-seq and qRT-PCR results showed that the expression profiles of nine out of ten genes were correlated between the qRT-PCR and RNA-seq (Table S10). The results of linear regression analysis indicated a correlation (*r*^2^ = 0.66) between the RNA-seq and qRT-PCR data (Figure S3).

## 3. Discussion

A total of 94 out of 363 markers showed polymorphism between JHN and RD6 (28.4%), which was lower than the polymorphism between IR64 and KDML105 (35.71%) from previous reports [32]. IR64 was developed by the International Rice Research Institute (IRRI), the Philippines, but KDML105 was developed by the Rice Department, Thailand. In this study, both JHN and RD6 are Thai rice varieties, therefore they may share a genetic background and show low levels of polymorphic markers. A higher polymorphic rate was found between rice from two subspecies, *Indica* and *Japonica* [33]. The 133 InDel markers used in this study were developed by the comparison between genome sequences of two different rice subspecies, *Indica* and *Japonica*. When applied to JHN and RD6, which are *Indica* rice, a low percentage (13.5%) of polymorphism was found [31].

Ninety-four polymorphic markers between JHN and RD6 were consequently used for graphical genotype construction of nine backcross inbred lines. The graphical genotyping illustrated that the genetic background of RD6 ranged from 92.55% to 96.81%. This result was fit with the expected percentage of the recurrent parent RD6 after 4 generations of backcrossing, with a hypothetical percentage of 96.875%. The RD0114 marker, a marker linked to a rice blast resistance QTL on chromosome 1, revealed a JHN background in all three qBL1 lines and all three qBL1&11 lines, but showed an RD6 background in all three qBL11 lines. This result indicated the successful introgression of the rice blast resistance QTL on chromosome 1. Similar to the three DNA markers (RM1233, RM224, and RM144) linked to a rice blast resistance QTL on chromosome 11 [27], all three markers revealed a JHN genetic background in all qBL1 lines and qBL1&11 lines, indicating the successful introgression of this QTL. 

From the pathogenicity assay, JHN and Q11 were resistant to a rice blast fungus THL84 isolate, but showed blast symptom with RD6 that was more severe than with Q1 (Figure 2). Our result was consistent with the previous report, showing that the *Pish*-containing rice line was partially resistant to the rice blast isolate THL84, while RD6 was susceptible to THL84. Moreover, the *Pik*-containing rice line and JHN showed resistance to the rice blast isolate THL84 [27].

Differentially expressed genes (DEGs) in the *Pish* and *Pik* backcross inbred lines and their gene function were identified and categorized into unique and shared sets. Identified DEGs uniquely present in the *Pish* rice line in this study were previously reported, including the wall-associated kinase gene involved in pathogen response [34], late embryogenesis abundant genes involved in desiccation and defense responses [35], and metallothionein-like protein regulation responses to stress stimuli and microbial challenge [36]. The identified DEGs uniquely present in the *Pik* rice line in this study were also previously reported, including zinc-finger-containing protein genes related to the regulation of resistance mechanisms for various biotic stress [37]; Myb transcription factor genes involved in controlling various processes, such as responses to biotic and abiotic stresses, development, differentiation, metabolism, and defense [38]; and chitinase genes involved in defense against fungal pathogens [39]. Finally, identified DEGs commonly present in both *Pish* and *Pik* rice lines were also previously reported, for example chitinase and substilin, which are associated with pathogen resistance [39,40].

Several identified DEGs were characterized based on their biotic stimulus (GO:0009607), defense response (GO:0006952), and defense response to fungus (GO:0050832). These defense response genes were highly expressed at 24 hai when compared with 0 hai. Pathogenesis-related protein class 10 (PR-10) plays a role as a ribonuclease. PR-10 was reported in several biotic stresses, including fungal pathogens [41]. Pathogenesis-related protein class 4 (PR-4) plays a role in mobilizing compounds during the senescence program and plays a protective role by degrading DNA or RNA of foreign invading pathogens [42]. PBZ1, another protein in the PR-10 family, was highly responsive to rice blast fungus infection and the reported RNase activity inside the cell [43]. Overall, our results showed that the *Pish* and *Pik* resistance genes share common signal transduction and defense response pathways in rice blast defense mechanisms, with few exceptions for specific genes in each case. 

The pathway analysis using Plant Reactome showed that brassinosteroids (BRs) are a class of steroid phytohormones regulating many aspects of plant growth and development. BRs are also reported to be involved in pathogen defense response [44]. In the brassinosteroid pathway, BKI1 (BRI1 kinase inhibitor1) is a negative regulator of brassinosteroid signaling [45]. Interestingly, brassinazole-resistant 1 protein (BZR1) showed high expression in both *Pish* and *Pik* rice lines at 24 hai. BZR1 is a nuclear component of the BR signal transduction pathway. BZR1 is a positive regulator of the BR signaling pathway, which mediates the downstream BR response and regulates BR biosynthesis [46]. Therefore, the resistance in the *Pish* and *Pik* rice lines may relate to the brassinosteroid signaling pathway. The higher expression level of the *BZR1* gene in the *Pik* rice line may correspond with the pathogenicity assay showing more resistance of the *Pik* rice line than the *Pish* rice line. Jasmonic acid (JA) signaling plays a role in plant defenses against pathogens [47]. The *Pik* rice line in our study showed high expression level of JA signaling genes *CM-LOX1* and *LOX2* lipoxygenase at 24 hai [48]. Moreover, Tify-domain-containing protein [49] and *WRKY* transcription factor genes [50], both of which are the JA signaling genes, were upregulated in the *Pik* rice line. Salicylic acid (SA) also plays an important role to induce plant defense against a variety of biotic and abiotic stresses [51]. In this study, pathogenesis-related protein *PRB1-2* [52], *BZIP* transcription factor [53], allergen *V5/Tpx-1*-related family protein [54] and pathogenesis-related 1a protein genes were upregulated. PR-1 is a dominant protein group reported to be induced by pathogens [55]. The validation of RNA-seq results using real-time PCR showed that the expression levels in nine out of ten selected genes were consistent with expression levels assessed by the RNA-seq. Many studies have used a real-time PCR to validate the gene expression profiles generated from the RNA-seq [22,56,57]. Our study also showed that the gene expression data from the RNA-seq could be validated by the real-time PCR, and the RNA-seq could indicate the reliability of the results of the gene expression levels. 

## 4. Materials and Methods

### 4.1. Rice Materials

Jao Hom Nin (JHN) is a non-glutinous rice variety that resists both leaf and neck blast diseases under natural conditions. RD6 is a commercial glutinous rice grown in the northeast region of Thailand, which is susceptible to various insect pests and diseases, including rice blast [27]. The development of RD6 backcross inbred lines introgressed by the rice-blast-resistant QTLs from JHN cultivar by marker-assisted selection was conducted at the Rice Science Center and Rice Gene Discovery Unit, Kasetsart University, Kamphaengsaen campus. The backcross inbred lines (BILs) containing a QTL from JHN were selected by markers linked to QTLs on chromosome 1 and 11. Nine BILs were obtained and classified into three groups, which consisted of three BILs with introgression of a rice blast resistance QTL on chromosome 1 of JHN (qBL1), three BILs with a rice blast resistance QTL on chromosome 11 of JHN (qBL11), and three BILs with both of these QTLs of JHN (qBL1&11). The rice line names were based on the group names, with the number of lines given in parentheses: qBL1 (1), qBL1 (2), qBL1(3), qBLl1 (1), qBLL11(2), qBL11(3), qBL1&11 (1), qBL1&11 (2), and qBL1&11 (3). KDML105 was used as a susceptible control in the pathogenicity assay.

### 4.2. Screening of Polymorphic Markers in JHN and RD6

To find polymorphic markers in JHN and RD6, InDel and simple sequence repeat (SSR) markers were used. Genomic DNA samples of JHN and RD6 rice cultivars were used for a PCR reaction to screen for polymorphic markers. Each PCR reaction component was filled in the 96-well plates. Then, 50 ng/μL DNA solution of each rice cultivar was used as the DNA template. The PCR reaction was prepared by mixing 2 μL of 50 ng/μL DNA, 0.5 μL of forward primer, 0.5 μL of reverse primer, 1 µL of 2.5 mM dNTP, 1 μL of 10× Buffer A, 0.5 μL of 50mM MgCl_2_, 0.15 μL of Taq DNA polymerase (Vivantis, CA, USA), and 4.35 μL of dH_2_O per 1 reaction. The PCR reaction was conducted by PCR machine (Eppendorf, Hamburg, Germany). The conditions for each step were as follows: 94 °C for 3 min for the predenaturation step, 94 °C for 45 s for the denaturation, 55 °C for 45 s for the annealing step, 72 °C for 1 min for the extension step, and 10 °C for 10 min for the postextension step. The denaturation to extension step was conducted over 35 cycles.

The primer pairs of 133 InDel markers [26] were used as primers to amplify DNA targets from DNA templates of JHN and RD6. The primer sequence information of InDel markers is shown in Table S1. The PCR products were visualized using 1.5% agarose gel electrophoresis. The 100 bp DNA ladder (Vivantis, CA, USA) was used to determine the sizes of PCR products in base pair units. For SSR markers, the primer pairs of 230 SSR markers were selected to study the polymorphism between JHN and RD6 rice cultivars. The primer sequence information of SSR markers is shown in Table S2. The PCR products were visualized by polyacrylamide gel electrophoresis. 

### 4.3. Graphical Genotyping

The polymorphic screening data generated from InDel and SSR markers of JHN, RD6, and nine BILs was used to construct the graphical genotype using GGT 2.0: Graphical GenoTyping [58]. In each marker, the PCR products of the same size as JHN were determined as the JHN genetic background, while the PCR products of the same size as RD6 were determined as the RD6 background.

### 4.4. Rice Blast Fungus Inoculation

The filter paper rice mycelium stock was used as the culture in the Petri dishes, containing rice flour agar (20 g rice flour, 28 g agar, and 2 g yeast extract in 1 L of dH_2_O) in an incubator at 28 °C for 7 days. The cultured fungus was recultured by cutting the 1 × 1 cm^2^ mycelial mat and transferring it to a Petri disc containing new rice flour agar for 7 days. The fungus was then scraped using a spreader and kept in a dry condition with black light exposure to induce conidia induction. The conidia suspension was prepared by washing the conidia in the Petri dishes using distilled water and preparing the 15 mL spore suspension. A hemocytometer was used to measure the conidia concentration. The conidia concentration was adjusted to 2 × 10^5^ conidia/mL by mixing with 0.01% Tween20. The spore suspension was sprayed onto 21-day-old rice seedlings. Rice plants were kept at 24 °C in high humidity (95–100% humidity) and dark conditions for 24 h. Rice leaf samples were collected at 0 and 24 h after inoculation for the transcriptomic study, while several rice plants were kept in the greenhouse for disease severity observation at 7 days post inoculation. The disease severity used a severity score ranging from 0 (resistance) to 6 (susceptibility), which measured the performance by lesion per unit area and lesion size [59].

### 4.5. Transcriptome Experiment and Data Analysis and Validation of Gene Expression Profiling

Three rice lines, namely RD6, a susceptible rice variety; Q1 BIL line with the *Pish* gene in RD6 genomic background; and Q11 BIL line with the *Pik* gene in RD6 genomic background, were used in the transcriptome experiment. A completely randomized design with two replications was used. The 21-day-old rice seedlings were grown in clay–loam soil in a cultivation tray. Each individual pot contained five plants, which were pooled and determined as one sample. The rice seedlings were inoculated by THL84, which can infect RD6, but this isolate cannot infect JHN, a resistant rice variety containing rice blast resistance genes *Pish* and *Pik* [30]. The pooled leaf samples of RD6, Q1, and Q11 lines were collected right before the inoculation. These samples were labelled as R-0, Q1-0, and Q11-0, respectively; and the leaves samples of RD6, Q1, and Q11 lines at 24 h after inoculation were collected and labelled as R-24, Q1-24, and Q11-24, respectively. The total RNA in each sample was isolated by GF-1 Total RNA Extraction Kit (Vivantis, CA, USA). Then, 5 μg of total RNA was subjected to library preparation using the True Standard mRNA Library. The quality and concentration of the RNA and library were assayed on a bioanalyzer (Agilent, CA, USA). The RNA integrity number (RIN) scores ranged from 8.00 to 8.60, which were sufficient quality for RNA-seq experiments (Table S9). Libraries were single-end sequenced by NextSeq 500/550 High-Output V2 Kit (Illumina, CA, USA).

After obtaining RNA-seq raw data, the sequencing data in FASTQ format were evaluated for quality using FASTQC. Low-quality bases were trimmed by Trimmomatic. High-quality single-end reads from each sample were aligned to the International Rice Genome Sequencing Project-1.0 (IRGSP-1.0) as the rice reference genome by using TopHat version 1.4.1 and assembled with Cufflinks. The cufflinks assemblies were merged together by Cuffmerge. Cuffdiff was used for identification of differentially expressed genes (DEGs), with false discovery rates (FDR) less than 0.05 between two samples and the expression level indicated by fragments per kilobase of transcript per million fragments (FPKM) value [60]. The expression levels were compared between RD6 and Q1, and between RD6 and Q11. Log_2_FC (binary logarithm of fold change) was calculated using the binary logarithm of the FPKM in 24 hai samples divided by the FPKM in 0 hai sample. The DEGs with Log_2_FC > 1 were determined as upregulated genes and with Log_2_FC < 1 were determined as downregulated genes. GO terms analysis of rice DEGs as enriched by agriGO v2.0 using the Rice Gramene locus as reference. The FDR significance level was 0.05 for GO enrichments using the hypergeometric method as the statistical test method and Yekutieli’s method (FDR under dependency) as the multitest adjustment method. A Venn diagram was built using Venny version 2.1. A heat map was displayed using heatmaply package in Rstudio software, using Z-scores that were transformed from FPKM values in each gene. The two different principal component analysis (PCA) plots were generated using the ggfortify package [61] in R studio, using FPKM values of ten randomly selected genes to analyze the association between samples and between biological replications to verify the correctness of sampling (Figure S4).

Quantitative RT-PCR was conducted using a one-step real-time PCR system and 100 ng of total RNA per reaction. Primers were designed using Primer3. The rice actin constitutively expressed gene was used as the reference gene. PCR amplification was performed in 10 µL of final contacting volumes of 2× QuantiFast SYBR Green Master Mix (Qiagen, Hilden, Germany). Three technical replicates for each of the two biological replicates were performed at 0 and 24 h after inoculation. Relative gene expression was calculated using the 2^−ΔΔCt^ method. 

## 5. Conclusions

Ninety-four polymorphic DNA markers between JHN and RD6 were used to construct the graphical genotypes of BC_4_F_2_ rice-blast-resistant Q1 and Q11 backcross inbred lines (BILs). The selected BILs with the highest percentage of RD6 genetic background, except for the QTLs on chromosome 1 and 11, were used for transcriptome analysis. RNA-seq results revealed 6466 and 6030 differentially expressed genes (DEGs) upon rice blast infection in the *Pish* and *Pik* rice lines, respectively. DEG analysis showed that rice blast resistance genes *Pish* and *Pik* have both unique and shared genes and pathways in defense response. Genes involved in recognition, signal transduction, and defense response were reported. Unique genes in the *Pish* rice line include wall-associated kinase gene and late embryogenesis abundant genes. Unique genes in the *Pik* rice line included chitinase gene and guanine nucleotide exchange factors for Rop gene. PR-4 and PR-10 protein genes were shared defense response genes in the *Pish* and *Pik* rice lines. Moreover, genes in brassinosteroid, jasmonic acid, and salicylic acid pathways were involved in defense response in the *Pish* and *Pik* rice lines. This study revealed that there are both unique and shared defense response genes involved in rice blast resistance of the *Pish* and *Pik* rice lines. The information obtained from this study will help to dissect and understand the defense response pathways in plants and will benefit rice-blast-resistant breeding programs worldwide.

## Figures and Tables

**Table 1:** Log_2_ (fold change of Fragments Per Kilobase Million value; FPKM value) of top 20 unique upregulated differentially expressed genes in Q1 and Q11.

**Unique to Q1**			
**No.**	**Gene ID**	**Gene Description**	**Log_2_(FC)**
1	OS06G0573500	Hypothetical conserved gene	6.439
2	OS11G0440866	Non-protein coding transcript	5.958
3	LEA3	Late embryogenesis abundant (LEA) protein	5.354
4	OS03G0573750	Conserved hypothetical protein	5.141
5	OS08G0190100	Germin-like protein 8-11, disease resistance	5.043
6	OS01G0899800	Pathogenesis-related transcriptional factor	4.965
7	OS09G0109600	Conserved hypothetical protein	4.552
8	OS04G0617900	Similar to germin-like protein subfamily 1	4.445
9	OS10G0180800	Wall-associated kinase	4.400
10	OS12G0571000	Metallothionein-like protein type 1	4.150
11	RAB16D	Dehydrin RAB 16D	3.984
12	OS12G0623400	Conserved hypothetical protein	3.839
13	OS03G0575500	Conserved hypothetical protein	3.826
14	OS01G0223000	Lipase	3.764
15	OS10G0418900	Hypothetical conserved gene	3.666
16	OS03G0223301	Hypothetical gene	3.490
17	OS01G0757200	GA 2-oxidase3, GA metabolism	3.482
18	OS10G0147400	Similar to Auxin influx carrier protein	3.300
19	OS02G0269650	Hypothetical gene	3.238
20	OS02G0627700	Similar to ATCNGC15	3.168
**Unique to Q11**			
**No.**	**Gene ID**	**Gene Description**	**Log_2_(FC)**
1	OS05G0454200	Guanine nucleotide exchange factors for Rop	6.649
2	OS09G0240200	Zinc finger, B-box domain containing protein	5.339
3	OS02G0514150	Hypothetical conserved gene	5.186
4	OS05G0261001	Non-protein coding transcript	4.767
5	EPlOSAG00000006021	n.a.	4.695
6	OS08G0335600	Protein of unknown function DUF568	4.681
7	OS02G0747900	Atypical basic helix–loop–helix protein	4.495
8	OS08G0486300	Similar to P-type R2R3 Myb protein	4.407
9	MADS5	MADS-box transcription factor	4.323
10	OS07G0600000	Conserved hypothetical protein	4.185
11	OS11G0134950	Hypothetical protein	4.090
12	OS02G0572000	Hypothetical protein	4.087
13	OS11G0701400	Chitinase (EC 3.2.1.14) III C10150-rice	4.069
14	OS04G0465300	A member of the GAST (gibberellin (GA)-Stimulated Transcript)	3.980
15	OS01G0725400	Uncharacterized protein family UPF0497	3.952
16	OS10G0107866	Non-protein coding transcript	3.926
17	OS06G0711900	Bifunctional inhibitor/plant lipid transfer protein	3.911
18	OS12G0157066	Hypothetical protein	3.809
19	OS10G0463200	Esterase, SGNH hydrolase-type domain containing protein.	3.764
20	OS01G0728100	Lipase, GDSL-domain-containing protein.	3.714

**Table 2:** Log_2_ (fold change of Fragments Per Kilobase Million value; FPKM value) of top 10 upregulated differentially expressed genes common between Q1 and Q11.

**Top 10 Upregulated DEGs of Q1**				
**No.**	**Gene ID**	**Gene Description**	**Log_2_FC**	
			**Q1**	**Q11**
1	OS10G0150800	Protein of unknown function DUF1210 family protein	6.738	6.007
2	OS02G0185900	Hypothetical conserved gene	6.532	6.011
3	OS10G0150700	Protein of unknown function DUF1210 family protein.	6.310	6.179
4	OS05G0375466	Non-protein coding transcript	6.152	7.593
5	OS08G0189850	Germin-like protein 8-9, disease resistance	5.874	5.836
6	OS09G0319800	Terpene synthase-like domain containing protein	5.842	8.216
7	OS04G0664900	Similar to H1005F08.5 protein	5.596	7.617
8	OS10G0150400	Protein of unknown function DUF1210 family protein	5.515	4.796
9	OS02G0827400	Similar to predicted protein	5.428	3.844
10	OS09G0315000	Similar to mpv17/PMP22 family protein	5.363	4.639
**Top 10 Upregulated DEGs of Q11**				
**No.**	**Gene ID**	**Gene Description**	**Log_2_FC**	
			**Q1**	**Q11**
1	OS09G0319800	Terpene synthase-like domain containing protein	5.842	8.216
2	OS11G0701500	Similar to class III chitinase homologue (*OsChib3H-g*)	3.739	7.891
3	OS04G0664900	Similar to H1005F08.5 protein	5.596	7.617
4	OS05G0375466	Non-protein coding transcript	6.152	7.593
5	OS10G0150700	Protein of unknown function DUF1210 family protein	6.31	6.179
6	OS02G0185900	Hypothetical conserved gene	6.532	6.011
7	OS10G0150800	Protein of unknown function DUF1210 family protein	6.738	6.007
8	OS08G0189850	Germin-like protein 8-9, disease resistance	5.874	5.836
9	OS07G0124900	Allergen V5/Tpx-1-related family protein	1.646	5.750
10	OS03G0291200	Protein of unknown function DUF231, plant-domain-containing protein	3.485	5.408

## Supplementary Materials

The following are available online at https://www.mdpi.com/2223-7747/9/6/694/s1, Table S1: Details of 133 rice InDel markers for screening of polymorphism between Jao Hom Nin (JHN) rice cultivar and RD6. Table S2: Details of 230 rice SSR markers for screening of polymorphism between Jao Hom Nin (JHN) rice cultivar and RD6. Table S3: List of 94 polymorphic markers between JHN and RD6. Table S4: Percentage of markers indicating JHN and RD6 genetic backgrounds in each rice backcross inbred line. Table S5: RNA-seq sample quality measured by bioanalyzer (Agilent 2100 Bioanalyzer system). TableS6: The list of upregulated DEGs in R-0 vs. Q1-0, and R-0 vs. Q11-0. Table S7: The number of upregulated genes grouped in each pathway by Plant Reactome database. Table S8: The number of downregulated genes grouped in each pathway by Plant Reactome database. Table S9: RNA integrity number (RIN) scores of twelve RNA samples in transcriptome analysis experiments. Table S10: The comparison of the Log_2_FC of expression values between RNA-seq and quantitative real-time PCR. Figure S1: Gene Ontology analysis indicated the top five GO terms of upregulated genes in Q1, Q11, and RD6 groups, based on the number of genes in each GO term. Figure S2: Gene Ontology analysis indicated the top five GO terms of downregulated genes in Q1, Q11, and RD6 groups, based on the number of genes in each GO term. Figure S3: Linear regression analysis indicated a correlation between the log2 (fold change) values from RNA-seq and qRT-PCR. Figure S4: PCA plot of twelve RNA-seq data sets according to gene expression (FPKM) level.
