# Supplementary material for: Transcriptome Comparison of Defense Responses in the Rice Variety ‘Jao Hom Nin’ Regarding Two Blast Resistant Genes, Pish and Pik
Source: Plants (Basel). 2020 May 29;9(6):694. doi: 10.3390/plants9060694 (PMC7356797; doi:10.3390/plants9060694)
Supplement: Supplementary file 1 [file plants-09-00694-s001.pdf]

Supplementary materials

Table 1. Details of 133 rice InDel markers for screening polymorphism between Jao Hom Nin (JHN) rice cultivar and RD6.

No.	Chr	Marker	Primer Sequences	
			Forward Primer (5´ → 3´)	Reverse Primer (5´ → 3´)
1	1	RD0101	GAGACCCGCCCTTACC	CCTTCACCCAGTGCTGC
2		RD0102	GCTACTTTGTGAATAAGGGTT	TTTGCCGCCTCTTGG
3		RD0103	CGACTTTCTTCTCGTCCC	TCAGCCATCGCTTTGC
4		RD0104	CTTCTGCCCTGGTCTAAA	AGCCTAAAACGGTCGTG
5		RD0105	AAACTCAATTCCCTCCGTC	CATCATGGGCAAGTAAGC
6		RD0106	AGGGGCGGTGCTGAT	GGTTAAAACCAAATGGATCA
7		RD0107	ATCACTTTCAACCCTACTCAG	TTAACATGACCAGTGCCTTA
8		RD0108	ATTAACCAAACATTCCCTAA	TACGCATGACAATAGCATAG
9		RD0109	AACCAAATTGACATGCTCT	TGCATAAAATCTCATAACCC
10		RD0110	AACACGAGGGCACGG	TGGGACGAAAGGAGTATG
11		RD0111	GGAGATCATCGTGGAGACC	TAGTTTGTTGCTTCCTACCG
12		RD0112	TTTGGGACTCTATTTTACTTTT	TGGGAGCAGGTCAACTAC
13		RD0113	AGGAGGAAGGGAGGGG	TGTCATTTGTCATCGTTGTG
14		RD0114	GCTTGTGGCAATTGGG	CGCCTGATGGATGTCG
15		RD0115	TCCAAAAGCGTCGTCA	TCCACTTCAGACCCAACTT
16		RD0116	GTAACCCCGCATCTTCT	TGTGCCAAACAAACCC
17		RD0117	TCCATGTGGCATCGAAT	TTTTAGGGAGTGAACGCA
18	2	RD0201	TAATTAGTGCAGCTGGAACT	ACTTAGAGGAAGAAGGAGGA
19		RD0202	CCAATTTGCATACGGAG	TCATCAGATATCAGAACGATC
20		RD0203	AGGTTTGCCGTTTTGTC	AGCGTCGCCAGGTG
21		RD0204	TAAAATGCATGGAGCATCA	TGTACAAGGTATACTCGTCTGG
22		RD0205	TTCTTTGATTGGCTTTGG	GTAGCATTTGAACTGGCATA
23		RD0206	TCTTTGATTGGCTTTGGT	GCATTTGAACTGGCATAAG
24		RD0207	GACGACAGATCCGAACG	GTGATACGCCAAAGGAAT
25		RD0208	GTAAGGCTACAATGATAAAA	CTCCAATCAAACGACC

Table S1. (Continued)

No.	Chr	Marker	Primer Sequences	
			Forward Primer (5′ → 3′)	Reverse Primer (5′ → 3′)
26	2	RD0209	GCCCTCCCCATTGC	CACGTTCGCTGTAGCC
27		RD0210	AGGCAGTAAGCCACC	TGTCACCTATGCTCCAA
28		RD0211	GTAGCACGAACTCCAAGAC	CTTAACAGAAATAACGGACAG
29		RD0212	AAATCCACCTTCATCCCT	AAGTACAAGTCCTTTCCATAGT
30		RD0213	TACCTTGCCGAATGCC	TGGTTCTGTGAGCCTGTG
31		RD0214	AGTTTTACCTTGGACGATTA	CAACCCGAGAATCCGT
32	3	RD0301	ACCGCCATCATCCAGA	GCATGCACTAGCAGCTTAG
33		RD0302	TTGAAGTTTTAGGAAATGCG	GGATCGAAGCTAGATGCTC
34		RD0303	GCGGCAAAGTCCAAAA	CGCTCTGAACTCACCAGAT
35		RD0304	GGCGTCACTGCTCGTA	GCCTGAAGCGTCCACA
36		RD0305	CACGGAGGTTGCTGTTG	CGACTCAAATATGGATCTATGT
37		RD0306	TGGAGCCCCACTAACAT	AAACAAACAAAGAACAAGCA
38		RD0307	GAGGAGGTGCCTACCAAG	TGCTTACAAGGGTCCAAT
39		RD0308	GTGAGGAGCAGACAAAGACT	AAGTGACATACAAAGAACGAG
40		RD0309	CGAGTTCTGAATTATACGGA	ACGCAACATCAGCACAG
41		RD0310	AGGATAAGACGAAGAGCACA	AAAAGGGGATCATCTCACT
42		RD0311	GGTGGCCCAGACGAT	AGAAAAAATGAGTTCC
43		RD0312	TTGTATTGCCTAAACGATCT	CATCCATCACGACCAGAA
44		RD0314	CCCAAGACAAGAGCAGACA	GTGGACGAGCAGCGAC
45	4	RD0401	GTAGGACTTACCCTTCGC	ATGATGTCCCACGCTTT
46		RD0402	TCGTGGACCAAGCACA	CGCAGAAAATGAAAGATATG
47		RD0403	GGTAAGCAAGCCAGCA	TTTAACTATTCGTCACTTTTAGG
48		RD0404	CCTGAGGAAAATGGGTT	TTGAATTTAGGGTGGATTAG
49		RD0405	GGAGCCCAGGAGCAA	TGCCTGTAACCAGTGCC
50		RD0407	AGAACTGCAAAGTAATGGGA	TGCTTATCTGGGAGGTGG

Table S1. (Continued)

No.	Chr	Marker	Primer Sequences	
			Forward Primer (5′ → 3′)	Reverse Primer (5′ → 3′)
51	4	RD0408	ATCGCCCAAAGTGCC	ACTCCCTCCGTTCAAAA
52		RD0409	TCAAGCACGCCACTCA	CCAACTCCGTCCACTAATA
53		RD0410	AGAAAGCGTGAAGAACGT	AAACAACAAGGGCGATG
54		RD0411	CATACCATACAGGCAACTAAA	CTGCTCATGTCTCGCTACT
55		RD0412	TCCGATGAGTCGTTTCTATC	ACTTTGCTGGGCTACTTG
56		RD0413	GGCAAATGAAGGATAGTCTC	TTTTCTGATGTGGCTCACT
57	5	RD0501	GTATTCGGTGGTGTAAGTGT	AATTTCCCTGTCCTGATG
58		RD0502	TCTAATTGGTTGGGCTCG	TGTTTTCTATCTTGTCCTTGC
59		RD0503	GGCGACGGAAGCAAAGC	CCAGCATCAAAACCAAAA
60		RD0504	CCCCTGGTTGGAACAC	TTAGGCCCATGCTGAGC
61		RD0506	GTTGGGAACCCACTCAC	AGAACTTGTCCCTCCTACAC
62		RD0507	TTAAGCACATCTTTTCGTTT	GCCAGGTGCAGATAGAGC
63		RD0508	CATGTTGATGAATAGCCT	ATCATCTAATGGGTGGG
64		RD0509	AATCGGGCTTGTGCTG	ACTTAAAATTCTTTTCGGACC
65		RD0510	CTCATGCAAAAGTTTTAGTGTA	ATTTCAACATCGGGAGAC
66		RD0511	CGTCCCTGTTGTTTAGACTTT	ACGGGGTGGGGTTAG
67		RD0512	TTGACGAAGGGGAACAT	AGGACTAGGACCAAGGCT
68	6	RD0601	GTTTGGGAGGGACGACT	CAAAATTTACTCTAGTTTATACTGC
69		RD0602	AAATAAAACCTTGAATCCCT	CCACTCATCTGCAAACCA
70		RD0603	CGTAAGATGTGGCTGAATG	CAAGTATAGACAGGTAGCAGGA
71		RD0605	TGGAAGTGGAGGCAACA	TTTTATCCCCAGAGGTCA
72		RD0607	AATACCAATCGTCATCAACA	GTCCAAAGTATGAGTAACAAGAA
73		RD0608	TGTACCTCGATTATCATCACATA	CCAGGCACTAATGCAACT
74		RD0610	ACTAATCAAGCCACTTCGG	AGTTTGGTCTGTCTCGGTC
75		RD0611	ATGGCACTCCTCTAGGTCA	GGCTTAGATTCGTTTCACAA

Table S1. (Continued)

No.	Chr	Marker	Primer Sequences	
			Forward Primer (5′ → 3′)	Reverse Primer (5′ → 3′)
76	6	RD0612	CTAGCTCCGTCATTTCCG	CATCCCAATATCCATCGTC
77		RD0613	AATCCCGAGCGATTAAC	GAACTAGGGCATAAGCAAG
78	7	RD0701	CAGAATCTCCAAGAAAACAA	TGCAACCACGTCACCTA
79		RD0702	GTTCCAGTCGTTGCTCG	AATCCATCGTATCACCAAAT
80		RD0703	TTAGGATAATTGCCTTCCA	GGAGTAGTGGTTATTGCTGAA
81		RD0704	ATCCTATTGAAAGCCCATC	ATCATTTTCTGCGAGGG
82		RD0705	TGGTAATTTGGACCTCAAG	CTGGGTGGTGGTATGTAAC
83		RD0706	GCTCAGGAACACCGATACC	CTTTCATACCTCATAGACCGT
84		RD0707	GCCAGCATTCTGAGATGA	GGCTCCCGTGAAGACC
85		RD0708	CTCTAATAAATGACAGGCTCTG	TCCTTTGTTACGTTGTGCTA
86		RD0709	CAGTGCCGGTCAAGACT	CCAATTTGATCCGTTCTAT
87		RD0710	CTGCTTCCTAAAACTTGCTA	CAAAGGACCTAAAGTGAACG
88		RD0711	AACGCGGTAGGAATAGG	CTGCATGGACCATACATAAT
89	8	RD0802	GCTCCTGTGGTTCCATG	CAGCGCAGCAGCTATTT
90		RD0804	GTGAGTCCGAGTCTGTCTTT	CGGCGACAATGGTGA
91		RD0805	CATGACAGGGGAAGAAGA	GCTAAAGTTAGAGCAAATCG
92		RD0806	GAGGTACACTCTGCCTGTG	GCTAACGACCGCCAAC
93		RD0807	CCAGTACCGTGTCCCC	ACCATCCTATGATAAAGGTGA
94		RD0808	CTGAACAGATACTATTCGAGATC	GTCGTGTCGCCCTACC
95		RD0809	CTGAAAGCATTTGGATACTCC	GATCAAGGTGCTCCGTC
96		RD0811	CACCCATTCGCAACAA	GGAGTGCGCCTATCCC
97		RD0812	CTTTCTCCCTGCGTCG	CAAATCACAAGCCCACTC
98	9	RD0901	GGTCGCCCCTTTGGT	GCTGCTTCCTTCTTCCCT
99		RD0902	TTATGACGCACATGCACTC	TCATGGCAATTTGGTCC
100		RD0904	TTCCAATTACCATCACTTCT	CAAAGGGTTGTTCCATTCT

Table S1. (Continued)

No.	Chr	Marker	Primer Sequences	
			Forward Primer (5′ → 3′)	Reverse Primer (5′ → 3′)
101	9	RD0905	TTGCTATTGATGTCATGCTT	ACTATGTTAGTTAGGTCCGTATT
102		RD0906	TTTGTGCCGATATGCC	TGCTATTTAAGTGCTTCCTAG
103		RD0907	TGTTAGTATGCTGAAGACGTG	CGATATGGAAGTTGTGGG
104		RD0908	CAGTATCAAGGATCTAAACGAT	AATAAGGGCAGCCAAAC
105		RD0909	AACCAAGGTCAGTTCATAGC	CACCTCGCTGCATGTCT
106		RD0910	CCGCAGCTTGGTCTCA	TCTGGTCACATCTGGAAGAC
107	10	RD1002	GCACCATAGGTTCTGCAT	CAAAACGCCCAGGAAT
108		RD1003	GTGAACCGCCGACAAC	AATCCCAAATCAACAAAGG
109		RD1004	GGCACTGCAACAACCC	CTAAACAGACCCAATATGACA
110		RD1006	TCACAATGGCTCTTCTCAT	GGGGCTTCACGCTGA
111		RD1007	ATACCCACCCGTCTCAA	TTCTTCCGTTACACGATGT
112		RD1008	GTGCAGCCCTCTTATCAA	GCCACAAATCGGTCGT
113		RD1009	AGTCCCCGTGCGCTAC	TGATGATGTCAATGCTCGTA
114	11	RD1101	CGTGGCGTGATACAC	ATTAGTCGCACATCCG
115		RD1103	TGGTTTGTCTGCCCCTAC	TCACCCCGTGCTGCTC
116		RD1104	TTGGTATTTGAAGCAGGTAT	GATACACCAACTATCTCCCA
117		RD1105	CGCACTACGAAACGGA	TTCAAGCATATCACAGAAGG
118		RD1106	AGGGTCTTTCCCATCACA	CTTTACAATTGGTATACGGTAA
119		RD1107	GCGGCGTCATTTATCC	TTGGGAGTTAGAGCAGGTA
120		RD1108	CTGTGGACCATTTGTCATT	ACCCTCCCGTACTCGC
121		RD1109	CGGAAAATGCTAAACAAA	ATGCTCTAGGTGCTGAATC
122		RD1110	GTCAGAGGAGTCGAATACG	ACCAACCAAACACCTAAAA
123		RD1111	TAGTACAATGAATCTGGACAGA	ACCCTTCACGCTCACTC
124	12	RD1201	TCTCCCCTCAAGAAACG	GTTGGAGGTAAGGCAAAG
125		RD1202	ATTCCATTCTTCTACTCGTCC	CCCTAGTTCCTGACCAAGTT

Table S1. (Continued)

No.	Chr	Marker	Primer Sequences	
			**Forward Primer (5′ → 3′)**	**Reverse Primer (5′ → 3′)**
126	12	RD1203	GAGTACGGACTACTGAATG	ATGCTGGATGAAACGA
127		RD1204	AGGATTGATGGGGCTTA	TGGGTTAGAGGTGGTTATG
128		RD1205	ATTTTCGGCGGAGTTAC	TCCACATGGCTTAGTTCATT
129		RD1207	GCCACTGCTAATAGGTTCA	TTCCGTTGCTATGGTCTC
130		RD1208	CGCCGCTCTTCCTCTT	GTGGACGCCGTTTGC
131		RD1209	CATCTCACCTCCCTACCAA	GGATGAACGCTCGGTT
132		RD1210	TTCACTGTAATAATCCGCCTTT	CCATCCCCAACCCACC
133		RD1211	TAACAACCACCTTGGATTT	CCACGAGCTGCGGACT

Table S2. Details of 230 rice SSR markers for screening polymorphism between Jao Hom Nin (JHN) rice cultivar and RD6.

No.	Marker	Chr.	Primer Sequences	
			Forward Primer (5′ → 3′)	Reverse Primer (5′ → 3′)
1	RM495	1	AATCCAAGGTGCAGAGATGG	CAACGATGACGAACACAACC
2	RM1		GCGAAAACACAATGCAAAAA	GCGTTGGTTGGACCTGAC
3	RM283		GTCTACATGTACCCTTGTTGGG	CGGCATGAGAGTCTGTGATG
4	RM259		TGGAGTTTGAGAGGAGGG	CTTGTTGCATGGTGCCATGT
5	RM312		GTATGCATATTTGATAAGAG	AAGTCACCGAGTTTACCTTC
6	RM5		TGCAACTTCTAGCTGCTCGA	GCATCCGATCTTGATGGG
7	RM129		TCTCTCCGGAGCCAAGGCGAGG	CGAGCCACGACGCGATGTACCC
8	RM237		CAAATCCCGACTGCTGTCC	TGGGAAGAGAGCACTACAGC
9	RM543		CTGCTGCAGACTCTACTGCG	AAATATTACCCATCCCCCCC
10	RM431		TCCTGCGAACTGAAGAGTTG	AGAGCAAAACCCTGGTTCAC
11	RM428		AACAGATGGCATCGTCTTCC	CGCTGCATCCACTACTGTTG
12	RM84		TAAGGGTCCATCCACAAGATG	TTGCAAATGCAGCTAGAGTAC
13	RM578		GGCGTCGTGTTTTCTCTCTC	CAAAAAGGAGGAGCAGATCG
14	RM34		GAAATGGCAATGTGTGCG	GCCGGAGAACCCTAGCTC
15	RM443		GATGGTTTTCATCGGCTACG	AGTCCCAGAATGTCGTTTCG
16	RM128		AGCTTGGGTGATTTCTTGGAAGCG	ACGACGAGGAGTCGCCGTGCAG
17	RM265		CGAGTTCGTCCAAGTGAGC	CATCCACCATTCCACCAATC
18	RM472		CCATGGCCTGAGAGAGAGAG	AGCTAAATGGCCATACGGTG
19	OSR23		TGATACGTGGTACGTGACGC	TAATCGCTTCCCTACCCCTG
20	RM414		ATTGCAGTCATGCAGCAGTC	ATATCTCCAATGTGGCAGGG
21	RM154	2	ACCCTCTCCGCCTCGCCTCCTC	CTCCTCCTCCTGCGACCGCTCC
22	RM53		ACGTCTCGACGCATCAATGG	CACAAGAACTTCCTCGGTAC

Table S2. (Continued)

No.	Marker	Chr.	Primer Sequences	
			Forward Primer (5′ → 3′)	Reverse Primer (5′ → 3′)
23	RM452	2	CTGATCGAGAGCGTTAAGGG	GGGATCAAACCACGTTTCTG
24	RM300		GCTTAAGGACTTCTGCGAACC	CAACAGCGATCCACATCATC
25	RM465		GTGCCTCCATCATCATCATC	TAGGACAAGCGAAGAAACCG
26	RM561		GAGCTGTTTTGGACTACGGC	GAGTAGCTTTCTCCCACCCC
27	RM106		CGTCTTCATCATCGTCGCCCCG	GGCCCATCCCGTCGTGGATCTC
28	RM6		GTCCCCTCCACCCAATTC	TCGTCTACTGTTGGCTGCAC
29	RM208		TCTGCAAGCCTTGTCTGATG	TAAGTCGATCATTGTGTGGACC
30	RM535		ACTACATACACGGCCCTTGC	CTACGTGGACACCGTCACAC
31	RM211		CCGATCTCATCAACCAACTG	CTTCACGAGGATCTCAAAGG
32	RM405		TCACACACTGACAGTCTGAC	AATGTGGCACGTGAGGTAAG
33	RM71		CTAGAGGCGAAAACGAGATG	GGGTGGGCGAGGTAATAATG
34	RM521		TTCCCTTATTCCTGCTCTCC	GGGATTTGCAGTGAGCTAGC
35	RM341		CAAGAAACCTCAATCCGAGC	CTCCTCCCGATCCCAATC
36	RM475		CCTCACGATTTTCCTCCAAC	ACGGTGGGATTAGACTGTGC
37	RM526		CCCAAGCAATACGTCCCTAG	ACCTGGTCATGACAAGGAGG
38	RM573		CCAGCCTTTGCTCCAAGTAC	TCTTCTTCCCTGGACCACAC
39	RM425		CCAACGAAGATTCGAAGCTC	CAGCACCATGAAGTCGCC
40	RM48		TGTCCCACTGCTTTCAAGC	CGAGAATGAGGGACAAATAACC
41	RM22	3	GGTTTGGGAGCCCATAATCT	CTGGGCTTCTTTCACTCGTC
42	RM489		ACTTGAGACGATCGGACACC	TCACCCATGGATGTTGTCAG
43	OSR13		CATTTGTGCGTCACGGAGTA	AGCCACAGCGCCCATCTCTC
44	RM251		GAATGGCAATGGCGCTAG	ATGCGGTTCAAGATTCGATC

Table S2. (Continued)

No.	Marker	Chr.	Primer Sequences	
			Forward Primer (5′ → 3′)	Reverse Primer (5′ → 3′)
45	RM338	3	CACAGGAGCAGGAGAAGAGC	GGCAAACCGATCACTCAGTC
46	RM16		CGCTAGGGCAGCATCTAAA	AACACAGCAGGTACGCGC
47	RM426		ATGAGATGAGTTCAAGGCCC	AACTCTGTACCTCCATCGCC
48	RM55		CCGTCGCCGTAGTAGAGAAG	TCCCGGTTATTTTAAGGCG
49	RM571		GGAGGTGAAAGCGAATCATG	CCTGCTGCTCTTTCATCAGC
50	RM514		AGATTGATCTCCCATTCCCC	CACGAGCATATTACTAGTGG
51	RM60		AGTCCCATGTTCCACTTCCG	ATGGCTACTGCCTGTACTAC
52	RM545		CAATGGCAGAGACCCAAAAG	CTGGCATGTAACGACAGTGG
53	OSR16		AAAACTAGCTTGCAAAGGGGA	TGCCGGCTGATCTTGTTCTC
54	RM7		TTCGCCATGAAGTCTCTCG	CCTCCCATCATTTCGTTGTT
55	RM563		CGACCCTAGGGTTTCTCC	CTCGACGTCGTGGAAAGC
56	RM282		CTGTGTCGAAAGGCTGCAC	CAGTCCTGTGTTGCAGCAAG
57	RM156		GCCGCACCCTCACTCCCTCCTC	TCTTGCCGGAGCGCTTGAGGTG
58	RM416		GGGAGTTAGGGTTTTGGAGC	TCCAGTTTCACACTGCTTCG
59	RM442		CTTAAGCCGATGCATGAAGG	ATCCTATCGACGAATGCACC
60	RM85		CCAAAGATGAAACCTGGATTG	GCACAAGGTGAGCAGTCC
61	RM307	4	GTACTACCGACCTACCGTTCAC	CTGCTATGCATGAACTGCTC
62	RM551		AGCCCAGACTAGCATGATTG	GAAGGCGAGAAGGATCACAG
63	RM518		CTCTTCACTCACTCACCATGG	ATCCATCTGGAGCAAGCAAC
64	RM471		ACGCACAAGCAGATGATGAG	GGGAGAAGACGAATGTTTGC
65	RM564		CATGGCCTTGTGTATGCATC	ATGCAGAGGATTGGCTTGAG
66	RM252		TTCGCTGACGTGATAGGTTG	ATGACTTGATCCCGAGAACG

Table S2. (Continued)

No.	Marker	Chr.	Primer Sequences	
			**Forward Primer (5′ → 3′)**	**Reverse Primer (5′ → 3′)**
67	RM303	4	GCATGGCCAAATATTAAAGG	GGTTGGAAATAGAAGTTCGGT
68	RM124		ATCGTCTGCGTTGCGGCTGCTG	CATGGATCACCGAGCTCCCCCC
69	RM280		ACACGATCCACTTTGCGC	TGTGTCTTGAGCAGCCAGG
70	RM559		ACGTACACTTGGCCCTATGC	ATGGGTGTCAGTTTGCTTCC
71	RM5688		GCAGTGTCCAACCATCTGTG	ATCTGGTCACCCTTTGCTTG
72	RM6314		GATTCGTGTCGGTTGTCAAG	GGTTCAGGGACGAATTTCAG
73	RM7396		GCTCCCTGCAACGAGATAAG	TAGAATCGCTGTGATCTGCG
74	RM3524		CGGAGCTGGTCTAGCCATC	GTCTCCGTCTTCCTCACTCG
75	RM1136		ATGTCATCCAGAGTCGCCTC	AGGACGTATTCACACACGAC
76	RM241		GAGCCAAATAAGATCGCTGA	TGCAAGCAGCAGATTTAGTG
77	RM317		CATACTTACCAGTTCACCGCC	CTGGAGAGTGTCAGCTAGTTGA
78	RM6748		ATTGGGTTTCTCATATTATG	CCAACACTCCTAACTAGTTC
79	RM3335		TAATCCACTGTGTCATTTAA	ACCATCATCTTGTACCTAGT
80	RM6441		CGAAAGGTCGGCATCTTTC	ATGGCAATGATACGGAGGAG
81	RM507	5	CTTAAGCTCCAGCCGAAATG	CTCACCCTCATCATCGCC
82	RM413		GGCGATTCTTGGATGAAGAG	TCCCCACCAATCTTGTCTTC
83	RM13		TCCAACATGGCAAGAGAGAG	GGTGGCATTCGATTCCAG
84	RM437		ACACCAACCAGATCAGGGAG	TGCTCGTCAATGGTGAGTTC
85	RM509		TAGTGAGGGAGTGGAAACGG	ATCGTCCCCACAATCTCATC
86	RM39		GCCTCTCTCGTCTCCTTCCT	AATTCAAACTGCGGTGGC
87	RM161		TGCAGATGAGAAGCGGCGCCTC	TGTGTCATCAGACGGCGCTCCG
88	RM178		TCGCGTGAAAGATAAGCGGCGC	GATCACCGTTCCCTCCGCCTGC

Table S2. (Continued)

No.	Marker	Chr.	Primer Sequences	
			Forward Primer (5′ → 3′)	Reverse Primer (5′ → 3′)
89	RM87	5	CCTCTCCGATACACCGTATG	GCGAAGGTACGAAAGGAAAG
90	RM334		GTTCAGTGTTCAGTGCCACC	GACTTTGATCTTTGGTGGACG
91	RM2010		ATCTTCTAGGAAATCGAGGA	GTTGGCAACTTGTAGTCTTG
92	RM7444		AATGGTACTACCGCCAGTGC	GGATTGTGATGTTCCTGAGG
93	RM5994		CAGGCACTGCAGCATGTC	AGGCTCGGCGAGATATTCTC
94	RM5140		GACGAGGTTGTTTATTAGTG	CTTATTTTCACGTGTACGTT
95	RM598		GAATCGCACACGTGATGAAC	ATGCGACTGATCGGTACTCC
96	RM459		CTGCAATGCTGCATGACC	CACTTTCTCTGCAGCACCAG
97	RM3663		CATCAACCTCCACGAACATG	CTCGGTGGTGATCCTCCTC
98	RM188		TCCGCCTCTCCTCTCGCTTCCC	GCAACGCACAACCGAACCGAGC
99	RM6313		ATCCAGATCCACTTTGACCG	GGAGGACTTCTACCATCCTTG
100	RM1054		TGCATATGTACCGCAACCTC	TTTCTGCATGATCCCCTCTG
101	RM133	6	TTGGATTGTTTTGCTGGCTCGC	GGAACACGGGGTCGGAAGCGAC
102	RM170		TCGCGCTTCTTCCTCGTCGACG	CCCGCTTGCAGAGGAAGCAGCC
103	RM510		AACCGGATTAGTTTCTCGCC	TGAGGACGACGAGCAGATTC
104	RM204		GTGACTGACTTGGTCATAGGG	GCTAGCCATGCTCTCGTACC
105	RM225		TGCCCATATGGTCTGGATG	GAAAGTGGATCAGGAAGGC
106	RM136		GAGAGCTCAGCTGCTGCCTCTAGC	GAGGAGCGCCACGGTGTACGCC
107	RM454		CTCAAGCTTAGCTGCTGCTG	GTGATCAGTGCACCATAGCG
108	RM162		GCCAGCAAAACCAGGGATCCGG	CAAGGTCTTGTGCGGCTTGCGG
109	RM400		ACACCAGGCTACCCAAACTC	CGGAGAGATCTGACATGTGG
110	RM141		CACCACCACCACCACGCCTCTC	TCTTGGAGAGGAGGAGGCGCGG

Table S2. (Continued)

No.	Marker	Chr.	Primer Sequences	
			**Forward Primer (5′ → 3′)**	**Reverse Primer (5′ → 3′)**
111	RM276	6	CTCAACGTTGACACCTCGTG	TCCTCCATCGAGCAGTATCA
112	RM6701		AGAGTGGCAAGAACAAATTC	ACAGATGCACTGATCCTGAC
113	RM8226		TTAGGATACGGCTTCTAGGC	CGTAATTGTTGCATATGGTG
114	RM564		CATGGCCTTGTGTATGCATC	ATGCAGAGGATTGGCTTGAG
115	RM7551		TCACCTCCTTCTGCCATCTC	CTAACTCACCTCCAGCCTGC
116	RM3827		GGACGGATTGTAGGTAGGAC	CCTTTCTTCAATCTGCATTC
117	RM7434		GGAGGAAAGGTTGGAGAAGG	TTTCCCGTATTCCATGAGCC
118	RM5371		GGCTAGCTTTAGCTGCGTTG	ACCCAGATCGAAACAACTGC
119	RM461		GAGACCGGAGAGACAACTGC	TGATGCGGTTTGACTGCTAC
120	RM494		GGGAGGGGATCGAGATAGAC	TTTAACCTTCCTTCCGCTCC
121	RM436	7	ATTCCTGCAGTAAAGCACGG	CTTCGTGTACCTCCCCAAAC
122	RM125		ATCAGCAGCCATGGCAGCGACC	AGGGGATCATGTGCCGAAGGCC
123	RM501		GCCCAATTAATGTACAGGCG	ATATCGTTTAGCCGTGCTGC
124	RM11		TCTCCTCTTCCCCCGATC	ATAGCGGGCGAGGCTTAG
125	RM10		TTGTCAAGAGGAGGCATCG	CAGAATGGGAAATGGGTCC
126	RM455		AACAACCCACCACCTGTCTC	AGAAGGAAAAGGGCTCGATC
127	RM234		ACAGTATCCAAGGCCCTGG	CACGTGAGACAAAGACGGAG
128	RM118		CCAATCGGAGCCACCGGAGAGC	CACATCCTCCAGCGACGCCGAG
129	RM172		TGCAGCTGCGCCACAGCCATAG	CAACCACGACACCGCCGTGTTG
130	RM428		AACAGATGGCATCGTCTTCC	CGCTGCATCCACTACTGTTG
131	RM295		CGAGACGAGCATCGGATAAG	GATCTGGTGGAGGGGAGG
132	RM4098		CGTTTGGATGAAGAAGAAGA	AGTGTTCGTTTCGGATTAGA
133	RM6872		GGATGAACACTGATGATGGC	ACCTCCACCACGATATCCAC

Table S2. (Continued)

No.	Marker	Chr.	Primer Sequences	
			**Forward Primer (5′ → 3′)**	**Reverse Primer (5′ → 3′)**
134	RM1253	7	CTGAACTTGCCTGAGAACTC	GACGACCTCTCCATGCTCG
135	RM7110		GGCGATCTCTGTGTTTATTG	ATTAACCGGTTGAGATGGTG
136	RM533		GCAACTGCTCTACGCCTCTC	CCTGAGGCTTCACCTACTCG
137	RM1973		GAGTTGCAAGGATATTTTAA	TGGAGCCTAGAGAATACATA
138	RM1132		ATCACCTGAGAAACATCCGG	CTCCTCCCACGTCAAGGTC
139	RM134		ACAAGGCCGCGAGAGGATTCCG	GCTCTCCGGTGGCTCCGATTGG
140	RM6650		AGCAGTCTCTGTTCGCGC	GACGACGACGACGATGACG
141	RM408	8	CAACGAGCTAACTTCCGTCC	ACTGCTACTTGGGTAGCTGACC
142	RM152		GAAACCACCACACCTCACCG	CCGTAGACCTTCTTGAAGTAG
143	RM25		GGAAAGAATGATCTTTTCATGG	CTACCATCAAAACCAATGTTC
144	RM544		TGTGAGCCTGAGCAATAACG	GAAGCGTGTGATATCGCATG
145	RM44		ACGGGCAATCCGAACAACC	TCGGGAAAACCTACCCTACC
146	RM404		CCAATCATTAACCCCTGAGC	GCCTTCATGCTTCAGAAGAC
147	RM339		GTAATCGATGCTGTGGGAAG	GAGTCATGTGATAGCCGATATG
148	RM284		ATCTCTGATACTCCATCCATCC	CCTGTACGTTGATCCGAAGC
149	RM433		TGCGCTGAACTAAACACAGC	AGACAAACCTGGCCATTCAC
150	RM447		CCCTTGTGCTGTCTCCTCTC	ACGGGCTTCTTCTCCTTCTC
151	RM38		ACGAGCTCTCGATCAGCCTA	TCGGTCTCCATGTCCCAC
152	RM2819		AATGTTGCTAGATTTAAAAC	CAGTAGGATATCTTACAACC
153	RM8243		CTCGTGCAACCATTATATTC	ACCTTAGCTGTCCTGAATTG
154	RM3153		CGGTTCTTTTCACATGGTCG	ATCACAACAAGCTCGACGTG
155	RM8264		ACGCTCCTCGCTTTCTAC	GCACCTCACACCAGTAATTC
156	RM195		AGAAAGAGAGGCCGTCGGCGGC	GGGCTCACCCCCAAACCTGCAG

Table S2. (Continued)

No.	Marker	Chr.	Primer Sequences	
			Forward Primer (5′ → 3′)	Reverse Primer (5′ → 3′)
157	RM556	8	ACTCCAAACCTCACTGCACC	TAGCACACTGAACAGCTGGC
158	RM256		GACAGGGAGTGATTGAAGGC	GTTGATTTCGCCAAGGGC
159	RM6948		GGTAAGTTGTCGGTTGCCTC	ACGTCCATACCAGGTCAAGC
160	RM3778		AGCTCGCGCAAGAGATGG	CCGCAAAAGTCTAGTTTGCC
161	RM316	9	CTAGTTGGGCATACGATGGC	ACGCTTATATGTTACGTCAAC
162	RM444		GCTCCACCTGCTTAAGCATC	TGAAGACCATGTTCTGCAGG
163	RM524		TGAAGAGCAGGAACCGTAGG	TCTGATATCGGTTCCTTCGG
164	RM105		GTCGTCGACCCATCGGAGCCAC	TGGTCGAGGTGGGGATCGGGTC
165	RM409		CCGTCTCTTGCTAGGGATTC	GGGGTGTTTTGCTTTCTCTG
166	RM410		GCTCAACGTTTCGTTCCTG	GAAGATGCGTAAAGTGAACGG
167	RM288		CCGGTCAGTTCAAGCTCTG	ACGTACGGACGTGACGAC
168	RM201		CTCGTTTATTACCTACAGTACC	CTACCTCCTTTCTAGACCGATA
169	RM215		CAAAATGGAGCAGCAAGAGC	TGAGCACCTCCTTCTCTGTAG
170	RM205		CTGGTTCTGTATGGGAGCAG	CTGGCCCTTCACGTTTCAGTG
171	RM3912		TGTGTGTGCCCGATCTAC	CCTCTCGATGAGCATTCC
172	RM5122		CTCGCAATTTATACGTAATC	CTCACGAAATAAAATGAGTG
173	RM3600		TGCCCACACATGATGAGC	AACGGGCAAGAGATCTTCTG
174	RM1553		AATTAGAGGGTCCACATGTC	ATTACCCTCATTTTCTACGC
175	RM6971		TTTGCGAACTAGACAAGGCC	GCGTCATTCTCGACGAGC
176	RM474	10	AAGATGTACGGGTGGCATTC	TATGAGCTGGTGAGCAATGG
177	RM222		CTTAAATGGGCCACATGCG	CAAAGCTTCCGGCCAAAAG
178	RM216		GCATGGCCGATGGTAAAG	TGTATAAAACCACACGGCCA
179	RM239		TACAAAATGCTGGGTACCCC	ACATATGGGACCCACCTGTC

Table S2. (Continued)

No.	Marker	Chr.	Primer Sequences	
			Forward Primer (5′ → 3′)	Reverse Primer (5′ → 3′)
180	RM467	10	GGTCTCTCTCTCTCTCTCTCTCTC	CTCCTGACAATTCAACTGCG
181	RM271		TCAGATCTACAATTCCATCC	TCGGTGAGACCTAGAGAGCC
182	RM258		TGCTGTATGTAGCTCGCACC	TGGCCTTTAAAGCTGTCGC
183	RM171		AACGCGAGGACACGTACTTAC	ACGAGATACGTACGCCTTTG
184	RM484		TCTCCCTCCTCACCATTGTC	TGCTGCCCTCTCTCTCTCTC
185	RM590		CATCTCCGCTCTCCATGC	GGAGTTGGGGTCTTGTTCG
186	RM7217		TTTGTAGGATGACACGTGGC	CGGGATTTCAGTACCTCACG
187	RM4455		CTCTCAAAGAACTAGGACTC	GAGAAGGTATGATAACCAAT
188	RM596		ATCTACACGGACGAATTGCC	AGAAGCTTCAGCCTCTGCAG
189	RM8202		AAGAATTGGTCAATAAGAGTGG	TTCTCTCTACCGGAGGATG
190	RM3123		ATTTCCCACACATCTCGCTG	GTGTCGCCGGTCAAGAAC
191	RM286	11	GGCTTCATCTTTGGCGAC	CCGGATTCACGAGATAAACTC
192	RM332		GCGAAGGCGAAGGTGAAG	CATGAGTGATCTCACTCACCC
193	RM552		CGCAGTTGTGGATTTCAGTG	TGCTCAACGTTTGACTGTCC
194	RM479		CCCCTTGCTAGCTTTTGGTC	CCATACCTCTTCTCCTCCCC
195	RM536		TCTCTCCTCTTGTTTGGCTC	ACACACCAACACGACCACAC
196	RM287		TTCCCTGTTAAGAGAGAAATC	GTGTATTTGGTGAAAGCAAC
197	RM457		CTCCAGCATGGCCTTTCTAC	ACCTGATGGTCAAAGATGGG
198	RM206		CCCATGCGTTTAACTATTCT	CGTTCCATCGATCCGTATGG
199	RM224		ATCGATCGATCTTCACGAGG	TGCTATAAAAGGCATTCGGG
200	RM144		TGCCCTGGCGCAAATTTGATCC	GCTAGAGGAGATCAGATGGTAGTGCATG
221	RM3483	12	CCTAGCTTTCAGGAGCAAG	CCCACAATGAGAAACAGTTG
222	RM491		ACATGATGCGTAGCGAGTTG	CTCTCCCTTCCCAATTCCTC

Table S2. (Continued)

No.	Marker	Chr.	Primer Sequences	
			Forward Primer (5′ → 3′)	Reverse Primer (5′ → 3′)
223	RM512	12	CTGCCTTTCTTACCCCCTTC	AACCCCTCGCTGGATTCTAG
224	RM2529		CATTAAAATCAGTGGGACTG	AGGCATTTCCTGATATGATC
225	RM1261		GTCCATGCCCAAGACACAAC	GTTACATCATGGGTGACCCC
226	RM309		GTAGATCACGCACCTTTCTGG	AGAAGGCCTCCGGTGAAG
227	RM463		TTCCCCTCCTTTTATGGTGC	TGTTCTCCTCAGTCACTGCG
228	RM1103		CAGCTGCTGCTACTACACCG	CTACTCCACGTCCATGCATG
229	RM3739		AGTTGCGCAGCTAATCGATC	AAGATCCAACGGGTTCTGTG
230	RM17		TGCCCTGTTATTTTCTTCTCTC	GGTGATCCTTTCCCATTTCA

Table S3. List of 94 polymorphic markers between JHN and RD6.

Chr.	Type	No. of Markers	Marker Name
1	InDel	3	RD0102, RD0109, RD0114
	SSR	3	RM1, RM495, RM5
2	InDel	2	RD0203, RD0211
	SSR	10	RM154, RM208, RM211, RM341, RM405, RM475, RM521, RM526, RM561, RM6
3	InDel	2	RD0305, RD0310
	SSR	7	OSR13, RM156, RM416, RM514, RM571, RM7, RM85
4	InDel	2	RD0401, RD0405
	SSR	9	RM241, RM252, RM280, RM303, RM317, RM417, RM564, RM5688, RM6748
5	InDel	0	
	SSR	5	RM13, RM161, RM188, RM5140, RM7444
6	InDel	1	RD0605
	SSR	10	RM136, RM170, RM225, RM276, RM314, RM400, RM402, RM510, RM564
7	InDel	0	
	SSR	5	RM234, RM295, RM501, RM6650, RM7110
8	InDel	0	
	SSR	7	RM152, RM223, RM25, RM404, RM408, RM433, RM515
9	InDel	3	RD0902, RD0904, RD0905
	SSR	2	RM524, RM1553
10	InDel	2	RD1002, RD1009
	SSR	5	RM216, RM222, RM271, RM590, RM8202
11	InDel	2	RD1108, RD1110, RD1233
	SSR	11	RM1233, RM144, RM1812, RM206, RM224, RM286, RM332, RM3717, RM5599, RM6091
12	InDel	1	RD1204
	SSR	2	RM17, RM309

Table S4. Percentage of markers indicating JHN and RD6 genetic background in each rice backcross inbred lines.

**Genetic Background**	**Lines**								
	**qBL1 (1)**	**qBL1 (2)**	**qBL1 (3)**	**qBL11 (1)**	**qBL11 (2)**	**qBL11 (3)**	**qBL1&11 (1)**	**qBL1&11 (2)**	**qBL1&11 (3)**
JHN	5.32%	3.19%	5.32%	5.32%	7.45%	5.32%	4.26%	7.45%	5.32%
RD6	94.68%	96.81%	94.68%	94.68%	92.55%	94.68%	95.74%	92.55%	94.68%

Table S5. RNA-seq sample quality measured by Bioanalyzer (Agilent 2100 Bioanalyzer system)

Samples	Total Reads	%GC	Total Mapped Reads	Multiple Alignment Reads
R-0-R1	13,933,704	50	13,020,280 (93.4%)	787,011 (6.0%)
Q1-0-R1	12,141,395	50	11,331,467 (93.3%)	690,690 (6.1%)
Q11-0-R1	13,939,379	50	13,030,257(93.5%)	783,049 (6.0%)
R-24-R1	14,609,308	50	13,634,250 (93.3%)	933,815 (6.8%)
Q1-24-R1	15,418,460	50	14,368,797 (93.2%)	949,706 (6.6%)
Q11-24-R1	14,066,771	50	13,158,619 (93.5%)	822,664 (6.3%)
R-0-R2	13,776,686	51	12,865,308 (93.4%)	790,190 (6.1%)
Q1-0-R2	11,724,286	51	10,930,304 (93.2%)	672,272 (6.2%)
Q11-0-R2	15,136,195	51	14,135,107 (93.4%)	873,434 (6.2%)
R-24-R2	13,868,768	51	12,858,754 (92.7%)	831,009 (6.5%)
Q1-24-R2	13,592,526	50	12,665,009 (93.2%)	821,962 (6.5%)
Q11-24-R2	13,649,979	51	12,674,416 (92.9%)	868,239 (6.9%)

TableS6. The list of up-regulated DEGs in R-0 vs Q1-0 and R-0 vs Q11-0.

		**R-0 vs Q1-0 set**	
**No.**	**Gene ID**	**Gene Description**	**Log_2FC**
1	OS02G0216300	Similar to cDNA clone:J023088C01, full insert sequence	4.85357
2	OS01G0781200	Similar to Rust resistance protein	4.48015
3	OS09G0357300	Hypothetical protein	4.46495
4	OS09G0358000	Similar to OsD305	4.44819
5	OS09G0425700	Similar to Glycylpeptide N-tetradecanoyltransferase	4.34583
6	OS05G0239900	Non-protein coding transcript	3.92151
7	OS01G0783800	Serine/threonine protein kinase-related domain containing protein	3.63835
8	OS02G0813200	Myb/SANT-like domain domain containing protein	3.25144
9	OS01G0144200	Vacuolar import and degradation protein Vid24 domain containing protein	3.18212
10	OS08G0231400	Germin-like protein 8-12, Disease resistance	2.67002
11	OS05G0325200	Cyclin-related 2 domain containing protein	2.48526
12	BURP16	beta subunit of polygalacturonase 1, Abiotic stress response	2.37316
13	OS08G0417000	2OG-Fe(II) oxygenase domain containing protein	1.83451
14	OS06G0647400	Similar to Lysosomal Pro-X carboxypeptidase	1.75392
15	OS03G0113000	Serine/threonine protein kinase domain containing protein	1.67333
16	OS02G0308800	FAS1 domain domain containing protein	1.66512
17	OS09G0424401	Hypothetical gene	1.6532
18	OS02G0140500	Similar to cycloartenol synthase	1.6524
19	OS05G0240200	Similar to NB-ARC domain containing protein	1.64474
20	OS04G0354600	Similar to Acyl CoA reductase-like protein	1.63535
21	OS03G0234900	Similar to Peroxidase	1.58841
22	OS01G0798100	Similar to cDNA clone:J023120H23, full insert sequence	1.55061

23	OS01G0908600	Proline (Pro) and γ-aminobutyric acid (GABA) transporter, Stress tolerance	1.51848
24	OS05G0202500	Conserved hypothetical protein	1.48955
25	OS04G0116600	Glucose/ribitol dehydrogenase family protein	1.47148
26	OS05G0456300	Aldose reductase	1.37337
27	BURP3	n.a.	1.34638
		**R-0 vs Q11-0 set**	
**No.**	**Gene ID**	**Gene Description**	**Log_2FC**
1	OS09G0358000	Similar to OSD305	4.55353
2	OS09G0357300	Hypothetical protein	4.43361
3	OS09G0425700	Similar to Glycylpeptide N-tetradecanoyltransferase	4.3851
4	OS07G0217600	CytochromeP450 monooxygenase, Diterpenoid phytoalexin biosynthesis, Bacterial blight resistance	3.83429
5	OS05G0239900	Non-protein coding transcript	3.52156
6	OS01G0144200	Vacuolar import and degradation protein Vid24 domain containing protein	3.26539
7	OS11G0660400	Hypothetical protein	3.15665
8	OS09G0255400	Similar to Indole-3-glycerol phosphate synthase	2.60061
9	OS11G0686500	NB-ARC domain containing protein	2.49103
10	OS08G0140300	Aromatic L-amino acid decarboxylase (AADC)	2.47502
11	OS04G0597700	Hypothetical protein	2.47385
12	OS05G0325200	Cyclin-related 2 domain containing protein	2.32238
13	OS03G0143400	Similar to mitochondrial chaperonin-60	2.13763
14	OS04G0354600	Similar to Acyl CoA reductase-like protein	2.13243
15	OS01G0106400	Similar to Isoflavone reductase homolog IRL	2.04085
16	OS04G0340800	Conserved hypothetical protein	2.03328
17	OS01G0382000	Similar to Pathogenesis-related protein PRB1-2 precursor	1.78951
18	OS11G0687100	von Willebrand factor, type A domain containing protein	1.75665
19	OS12G0268100	Hypothetical protein	1.72806
20	OS01G0723600	Ribose-phosphate pyrophosphokinase 3 (EC 2.7.6.1) (Phosphoribosyl pyrophosphate synthetase 3)	1.70517
21	OS05G0202500	Conserved hypothetical protein	1.66019
22	OS03G0234900	Similar to Peroxidase	1.6569
23	OS08G0331800	Conserved hypothetical protein	1.63905
24	OS06G0647400	Similar to Lysosomal Pro-X carboxypeptidase	1.63846
25	OS05G0456300	Aldose reductase	1.60436
26	OS09G0424401	Hypothetical gene	1.59364
27	OS11G0644700	Plant disease resistance response protein family protein	1.57546
28	OS04G0630300	NAD(P)-binding domain containing protein	1.43573
29	OS05G0240200	Similar to NB-ARC domain containing protein, expressed	1.42657
30	OS04G0209200	Similar to MRP-like ABC transporter	1.41845
31	PPDK1	Cytosolic pyruvate orthophosphate dikinase	1.33577

32	OS03G0264400	Anthranilate synthase alpha 2 subunit	1.30608

Table S7. The number of up-regulated genes grouped in each pathway by Plant reactome database.

No.	Pathway Name	No. of Genes		
		Q1 Set	Q11 Set	RD6 Set
1	13-LOX and 13-HPL pathway	2	2	2
2	Alanine biosynthesis II			1
3	Allantoin degradation			2
4	Amine and polyamine biosynthesis	3	3	3
5	Amino acid biosynthesis	13	11	23
6	Amino acid catabolism	15	15	18
7	Amino acid metabolism	27	26	39
8	Aminopropanol biosynthesis	1	2	1
9	Arginine biosynthesis	1	1	1
10	Arginine degradation	1	1	1
11	Arsenic uptake and detoxification		1	2
12	Ascorbate biosynthesis	2	2	2
13	Asparagine biosynthesis	1	1	2
14	Asparagine degradation I			1
15	Aspartate biosynthesis I			1
16	Auxin signalling	3	5	2
17	Auxin transport	2	4	4
18	Beta-alanine biosynthesis I	1	1	1
19	Beta-alanine biosynthesis II	1	1	2
20	Beta-alanine biosynthesis III			2
21	Biotin biosynthesis II	2	1	4
22	Brassinosteroid biosynthesis II	1	1	1
23	Brassinosteroid signaling	3	4	5
24	Calvin cycle	2	1	3
25	Carbohydrate metabolism	13	11	17
26	Cell cycle		1	
27	Cellular processes		1	
28	Chlorophyll a biosynthesis II	1	1	1
29	Choline biosynthesis I	1	1	3
30	Chorismate biosynthesis	2	1	2
31	Circadian rhythm	4	4	4
32	Citrulline biosynthesis	2	1	2
33	Cofactor biosyntheses	10	8	17
34	Cyanate degradation	1	1	1
35	Cysteine biosynthesis I	1	1	1
36	Cytokinins-O-glucoside biosynthesis	3	1	2
37	Cytosolic glycolysis	1	1	2
38	Detoxification	1	1	1
39	Development of root hair		1	1
40	Divinyl ether biosynthesis II (13-LOX)	2	2	2
41	Ent-kaurene biosynthesis	1	1	
42	Ethylene biosynthesis and signaling	2	2	1
43	Ethylene biosynthesis from methionine	2	2	1

44	Fatty acid and lipid metabolism	1	2	9
45	Flavonoid biosynthesis	1		1
46	Fructan biosynthesis		1	1
47	G1 phase		1	
48	GA12 biosynthesis	1	2	1
49	Galactose degradation II	1	1	3
50	Galactosylcyclitol biosynthesis			1
51	Gamma-glutamyl cycle			1
52	GDP-mannose metabolism	2	2	2
53	Generation of precursor metabolites and energy	2	2	3
54	Generation of superoxide radicals			1
55	Geranyldiphosphate biosynthesis			1
56	Geranylgeranyldiphosphate biosynthesis II (plastidic)			1
57	Gibberellin signaling	1	2	2
58	Glutamate biosynthesis I			1
59	Glutamate degradation	1	1	1
60	Glutathione biosynthesis			1
61	Glycerol degradation I	1	1	1
62	Glycolipid desaturation		1	
63	Growth and developmental processes	11	13	14
64	Hormone signaling, transport, and metabolism	28	33	42
65	Hydroxycinnamic acid serotonin amides biosynthesis			1
66	IAA biosynthesis I	1	1	3
67	Inflorescence development	1	1	1
68	Inorganic nutrients metabolism	7	7	6
69	Intracellular auxin transport	1	2	2
70	Iron uptake and transport in root vascular system	3	3	3
71	Isoleucine biosynthesis from threonine			1
72	Jasmonic acid biosynthesis	2	2	2
73	Jasmonic acid signaling	3	4	9
74	Kievitone biosynthesis			1
75	Leucine biosynthesis	3	3	3
76	Long day regulated expression of florigens	1	1	1
77	Lysine degradation I	1	1	
78	Lysine degradation II	2	2	3
79	Mannose degradation	2	2	2
80	Metabolism and regulation	89	95	146
81	Methionine salvage pathway	2	3	2
82	Methylerythritol phosphate pathway	2	1	2
83	Mevalonate pathway	1	1	2
84	Mitosis		1	
85	Momilactone biosynthesis	1	3	1
86	Mugineic acid biosynthesis	3	3	2
87	Myo-inositol biosynthesis	1	1	1
88	NAD biosynthesis I (from aspartate)			1
89	Nucleotide metabolism	2	2	5
90	Ornithine biosynthesis	1	1	1
91	Oryzalexin S biosynthesis			1

92	Pantothenate and coenzyme A biosynthesis III	1		3
93	Pantothenate biosynthesis I	1		1
94	Pantothenate biosynthesis II	1		1
95	Pantothenate biosynthesis III	1		1
96	Phaseic acid biosynthesis		1	1
97	Phenylalanine biosynthesis I	1	1	3
98	Phenylalanine degradation III	1	1	1
99	Phenylpropanoid biosynthesis	2	2	3
100	Phenylpropanoid biosynthesis, initial reactions		1	4
101	Phospholipid biosynthesis I			3
102	Photorespiration	1		2
103	Phytic acid biosynthesis	1	2	3
104	Phytic acid biosynthesis (lipid-independent)	1	2	3
105	Phytyl-PP biosynthesis	1	1	1
106	Polar auxin transport	1	2	2
107	Polyisoprenoid biosynthesis			1
108	Proline biosynthesis V (from arginine)	1		1
109	Proline degradation	3	3	3
110	PRPP biosynthesis I	1	1	2
111	Putrescine biosynthesis II	1	1	1
112	Pyridoxal 5'-phosphate biosynthesis	1		1
113	Reactive oxygen species (ROS) homeostasis			1
114	Regulation of embryo development	1		
115	Regulation of leaf development	2	1	3
116	Regulation of seed size	6	10	8
117	Regulatory network of nutrient accumulation	1	1	2
118	Reproductive structure development	9	12	11
119	Response to cold temperature	3	2	3
120	Response to Drought	2	2	2
121	Response to heavy metals	1		2
122	Response to iron deficiency	6	6	5
123	Response to salinity	2	2	2
124	Response to submergence	2	1	1
125	Responses to stimuli: abiotic stimuli and stresses	7	6	8
126	Root structure development		1	1
127	Root-specific gene network of NAC10_TF induced by drought, salinity, and ABA	1	1	1
128	S-adenosyl-L-methionine cycle	1	1	
129	Salicylate biosynthesis		1	4
130	Salicylic acid signaling	6	7	11
131	Secologanin and strictosidine biosynthesis			1
132	Secondary metabolism	9	14	21
133	Seed development	8	11	10
134	Short day regulated expression of florigens	1	1	1
135	SNAC1 transcription network involved in drought and salinity tolerance	1	1	1
136	Spermidine biosynthesis	2	2	
137	Spermine biosynthesis	2	2	

138	Sphingolipid metabolism			3
139	Stachyose biosynthesis	1	1	1
140	Starch biosynthesis	1	1	2
141	Strigolactone signaling	1	1	2
142	Suberin biosynthesis		1	4
143	Sucrose biosynthesis	3	2	1
144	TCA cycle (plant)	2	2	3
145	Thiamine biosynthesis	1		1
146	Threonine degradation	1	1	1
147	Trans,trans-farnesyl diphosphate biosynthesis			1
148	Trehalose biosynthesis I	1	1	1
149	Trehalose degradation II	1	1	1
150	Tryptophan biosynthesis			1
151	Tyrosine degradation I	4	4	5
152	UDP-D-glucuronate biosynthesis (from myo-inositol)	1	1	1
153	Underwater shoot and internode elongation	2	1	1
154	Ureide biosynthesis	1	1	3
155	Valine biosynthesis			1
156	Valine degradation	4	4	5
157	Vegetative structure development	2	1	3
158	Vitamin E biosynthesis	1	1	1
159	Wighteone and luteone biosynthesis			1
160	Xylogalacturonan biosynthesis	1		
161	Xyloglucan biosynthesis			1

Table S8. The number of down-regulated genes grouped in each pathway by Plant reactome database.

No.	Pathway Name	No. of Genes		
		**Q1 Set**	**Q11 Set**	**RD6 Set**
1	13-LOX and 13-HPL pathway	1	1	
2	Activation of pre-replication complex	7	4	3
3	Acyl-CoA synthetase pathway		1	
4	Alanine biosynthesis II		1	
5	Allantoin degradation	1	3	1
6	Amine and polyamine biosynthesis	1	3	1
7	Amino acid biosynthesis	33	39	25
8	Amino acid catabolism	13	12	11
9	Amino acid metabolism	43	48	32
10	Aminopropanol biosynthesis	1	2	3
11	Ammonia assimilation cycle	2	2	2
12	Anther and pollen development	1	1	1
13	Arginine biosynthesis	1	1	1
14	Arsenic uptake and detoxification	4	4	5
15	Ascorbate biosynthesis	4	5	4
16	Asparagine biosynthesis	2	2	2
17	Asparagine degradation I	3	2	1
18	Aspartate biosynthesis I	1	1	1
19	Assembly of pre-replication complex	3	2	2
20	Auxin signalling	13	9	9

21	Auxin transport	1		1
22	Beta-alanine biosynthesis I	2	2	
23	Beta-alanine biosynthesis II	1	1	1
24	Brassinosteroid biosynthesis II	1	1	1
25	Brassinosteroid signaling	4	3	3
26	Calvin cycle	9	10	10
27	Canavanine biosynthesis	1	1	1
28	Carbohydrate metabolism	41	41	32
29	Cardiolipin biosynthesis	1	2	2
30	Carotenoid biosynthesis	2	1	2
31	Cell cycle	10	7	6
32	Cellular processes	10	7	6
33	Cellulose biosynthesis	5	3	3
34	Chlorophyll a biosynthesis I	1	1	1
35	Chlorophyll cycle	1	1	1
36	Cholesterol biosynthesis I	1	1	1
37	Cholesterol biosynthesis II (via 24,25-dihydrolanosterol)		1	1
38	Cholesterol biosynthesis III (via desmosterol)	1	1	1
39	Choline biosynthesis I	2	2	2
40	Choline biosynthesis III	4	4	3
41	Chorismate biosynthesis	3	3	2
42	Circadian rhythm	4	3	4
43	Citrulline biosynthesis	1	1	1
44	CMP-KDO biosynthesis II (from D-arabinose 5-phosphate)	1	1	
45	Cofactor biosyntheses	19	22	17
46	Cyanate degradation	2	3	2
47	Cysteine biosynthesis I	2	2	1
48	Cysteine degradation	1		
49	Cytokinins 7-N-glucoside biosynthesis	1	1	1
50	Cytokinins 9-N-glucoside biosynthesis	1	1	1
51	Cytokinins-O-glucoside biosynthesis	3	3	2
52	Cytosolic glycolysis	3	3	3
53	Detoxification	7	7	6
54	Development of root hair	2	2	1
55	Divinyl ether biosynthesis II (13-LOX)	1	1	
56	Dolichyl-diphosphooligosaccharide biosynthesis	1		
57	Ethylene biosynthesis and signaling	1	1	
58	Ethylene biosynthesis from methionine	1	1	
59	Fatty acid and lipid metabolism	18	19	12
60	Flavin biosynthesis	3	3	2
61	Flavonoid biosynthesis	5	5	4
62	Folate polyglutamylation I	3	3	2
63	Folate polyglutamylation II	2	2	2
64	Fructan biosynthesis	1	1	
65	G1 phase	2	1	2
66	G1/S transition	10	7	6

67	Galactose degradation II	4	4	2
68	GDP-D-rhamnose biosynthesis	2	2	1
69	GDP-L-fucose biosynthesis I (from GDP-D-mannose)	2	2	1
70	GDP-mannose metabolism	1	1	1
71	Generation of precursor metabolites and energy	1	1	1
72	Generation of superoxide radicals	5	5	3
73	Gentiodelphin biosynthesis	1		
74	Gibberellin signaling	3	2	1
75	Glutamate biosynthesis I	2	2	
76	Glutamate biosynthesis IV	1	1	1
77	Glutamate biosynthesis V	1	1	1
78	Glutamate degradation	1	3	3
79	Glutamine biosynthesis I	1	1	1
80	Glutathione redox reactions I	1	1	1
81	Glutathione redox reactions II	1	1	1
82	Glycine betaine biosynthesis III		1	
83	Glycolipid desaturation	3	3	1
84	Growth and developmental processes	40	36	30
85	Histidine biosynthesis I	1	2	3
86	Homoserine biosynthesis	4	3	3
87	Hormone signaling, transport, and metabolism	55	44	31
88	HSFA7/ HSFA6B-regulatory network-induced by drought and ABA.	1	1	1
89	Hydroxycinnamic acid serotonin amides biosynthesis	3	4	3
90	IAA biosynthesis I	6	7	5
91	IAA biosynthesis II	1		
92	IAA conjugate biosynthesis I	1		
93	Inflorescence development	2	2	2
94	Inorganic nutrients metabolism	8	8	7
95	Isoleucine biosynthesis from threonine	2	2	1
96	Jasmonic acid biosynthesis	5	5	2
97	Jasmonic acid signaling	15	11	7
98	Lactucaxanthin biosynthesis	1		1
99	Leucodelphinidin biosynthesis	1	1	1
100	Leucopelargonidin and leucocyanidin biosynthesis	1	1	1
101	Linear furanocoumarin biosynthesis	1	1	1
102	Lipid-A-precursor biosynthesis	1	1	1
103	Long day regulated expression of florigens	2	2	2
104	Lysine biosynthesis I	6	5	5
105	Lysine biosynthesis II	6	5	5
106	Lysine biosynthesis VI	5	4	4
107	Lysine degradation I	1	1	
108	Lysine degradation II		1	1
109	Maackiain biosynthesis	2		
110	Mannose degradation	1	1	1
111	Medicarpin biosynthesis	2		
112	Metabolism and regulation	212	212	152
113	Methionine biosynthesis II	2	2	2

114	Methionine salvage pathway	1	1	
115	Methylerythritol phosphate pathway	2	1	2
116	Mevalonate pathway	2	3	
117	Mitochondrial pyruvate metabolism	1	1	1
118	Mitosis	10	7	6
119	Nitrate assimilation	3	2	2
120	Nucleotide metabolism	1	3	1
121	Peptidoglycan biosynthesis I	2	2	2
122	Phaseic acid biosynthesis	1	1	1
123	Phenylalanine biosynthesis I	3	3	3
124	Phenylalanine degradation III	4	4	4
125	Phenylpropanoid biosynthesis	4	3	2
126	Phenylpropanoid biosynthesis, initial reactions		1	
127	Phospholipid biosynthesis I	5	4	2
128	Photorespiration	12	13	8
129	Phytic acid biosynthesis	1	1	1
130	Phytic acid biosynthesis (lipid-independent)	1	1	1
131	Pinobanksin biosynthesis	2	2	2
132	Plastid glycolysis	3	3	3
133	Polar auxin transport	1		1
134	Polyisoprenoid biosynthesis	1		
135	Proline degradation		1	1
136	PRPP biosynthesis I		1	
137	Reactive oxygen species (ROS) homeostasis	7	7	6
138	Recognition of fungal and bacterial pathogens and immunity response	4	2	2
139	Regulation of embryo development	1	1	
140	Regulation of leaf development	3	3	2
141	Regulation of seed size	20	15	15
142	Regulatory network of nutrient accumulation	8	9	7
143	Removal of superoxide radicals	2	2	3
144	Reproductive meristem phase change	4	4	3
145	Reproductive structure development	35	31	27
146	Response to Aluminum stress	1	1	1
147	Response to cold temperature	3	2	1
148	Response to Drought	1	1	1
149	Response to heavy metals	5	5	6
150	Response to phosphate deficiency	2	1	1
151	Responses to stimuli: abiotic stimuli and stresses	10	8	8
152	Responses to stimuli: biotic stimuli and stresses	4	2	2
153	Root structure development	2	2	1
154	Salicylate biosynthesis	2		1
155	Salicylic acid signaling	9	9	5
156	Secologanin and strictosidine biosynthesis	2	3	2
157	Secondary metabolism	26	25	18
158	Seed development	29	25	22
159	Short day regulated expression of florigens	1	1	1
160	Sphingolipid metabolism	2	2	1

161	Starch biosynthesis	6	6	6
162	Sterol biosynthesis	1	1	1
163	Strigolactone biosynthesis			1
164	Strigolactone signaling	3	3	3
165	Suberin biosynthesis	1	2	1
166	Sucrose biosynthesis	5	5	4
167	TCA cycle (plant)	1	1	1
168	Tetrahydrofolate biosynthesis I	1	3	4
169	Tetrahydrofolate biosynthesis II	4	5	2
170	Thiosulfate disproportionation III (rhodanese)	1	1	1
171	Threonine biosynthesis from homoserine	1	1	
172	Threonine degradation	1	1	1
173	Trans-zeatin biosynthesis	1	1	
174	Trehalose degradation II	1	2	1
175	Tricin biosynthesis	3	3	2
176	Tryptophan biosynthesis	2	6	1
177	Tyrosine degradation I	3	3	3
178	UDP-D-apiose biosynthesis	1	1	1
179	UDP-D-xylose biosynthesis	3	3	2
180	Ureide biosynthesis	1	2	1
181	Valine biosynthesis	2	2	1
182	Vegetative structure development	5	5	3
183	Vitamin E biosynthesis	2	2	1
184	Xylan biosynthesis	2	3	1
185	Xylogalacturonan biosynthesis	2	2	1
186	Xylose degradation	1	1	1

Table S9. RNA integrity number (RIN) score of twelve RNA samples in transcriptome analysis experiment.

**Sample**	**RIN Score**	**Sample**	**RIN Score**
R-0-R1	8.30	R-0-R2	8.40
Q1-0-R1	8.50	Q1-0-R2	8.20
Q11-0-R1	8.60	Q11-0-R2	8.30
R-24-R1	8.20	R-24-R2	8.00
Q1-24-R1	8.40	Q1-24-R2	8.00
Q11-24-R1	8.50	Q11-24-R2	8.30

Table S10. The comparison of Log_2FC of expression value between RNA-seq and quantitative real-time PCR.

**No.**	**Gene ID**	**Gene Description**	**RNA-seq**		**qRT-PCR**	
			**Q1-24 vs Q1-0**	**Q11-24 vs Q11-0**	**Q1-24 vs Q1-0**	**Q11-24 vs Q11-0**
1	OS06G0573500	Hypothetical conserved gene	6.44		1.93	1.83
2	OS10G0180800	Wall-associated kinase	4.4		1.32	0.00
3	OS10G0504900	Similar to Lipid transfer protein	4.07		0.03	1.75
4	OS02G0514150	Hypothetical conserved gene		5.19	0.41	0.94
5	OS11G0134950	Hypothetical protein		4.09	1.90	1.47
6	OS11G0701400	Chitinase (EC 3.2.1.14)		4.07	1.12	-0.25
7	OS07G0529000	Similar to Isocitrate	8.28	6.5	4.35	4.24

		lyase				
8	OS08G0157600	MYB transcription factor, Circadian clock	7.82	7.6	3.35	3.90
9	OS09G0319800	Terpene synthase-like domain containing protein	5.84	8.22	2.14	2.62
10	OS11G0701500	Similar to Class III chitinase homologue	3.74	7.89	1.91	5.33

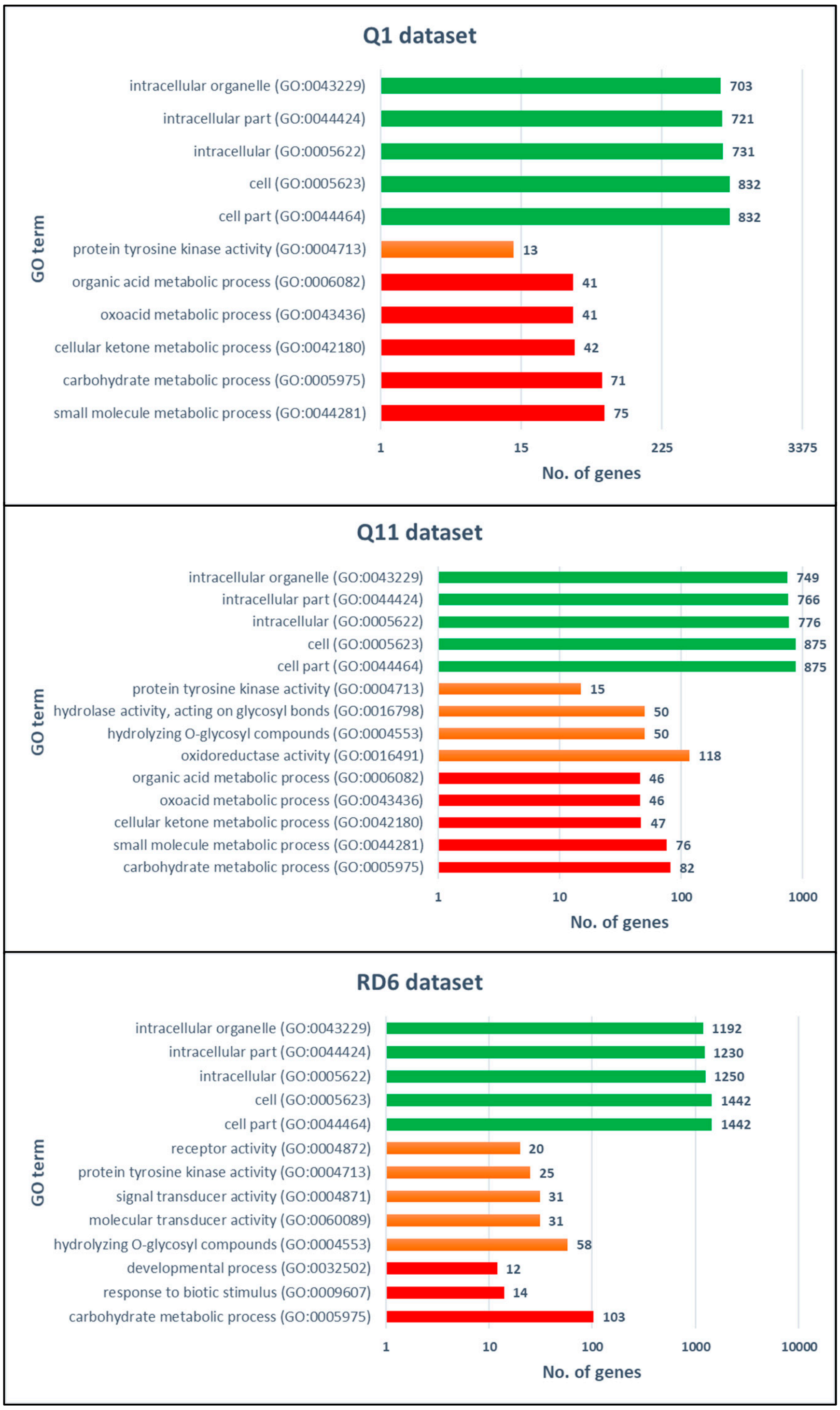

Figure S1. gene ontology analysis indicated the top five GO terms of up-regulated genes in Q1, Q11 and RD6 group, based on the number of genes in each GO term. Note: Biological process (red bar), Molecular function (orange bar) and Cellular

component (green bar)

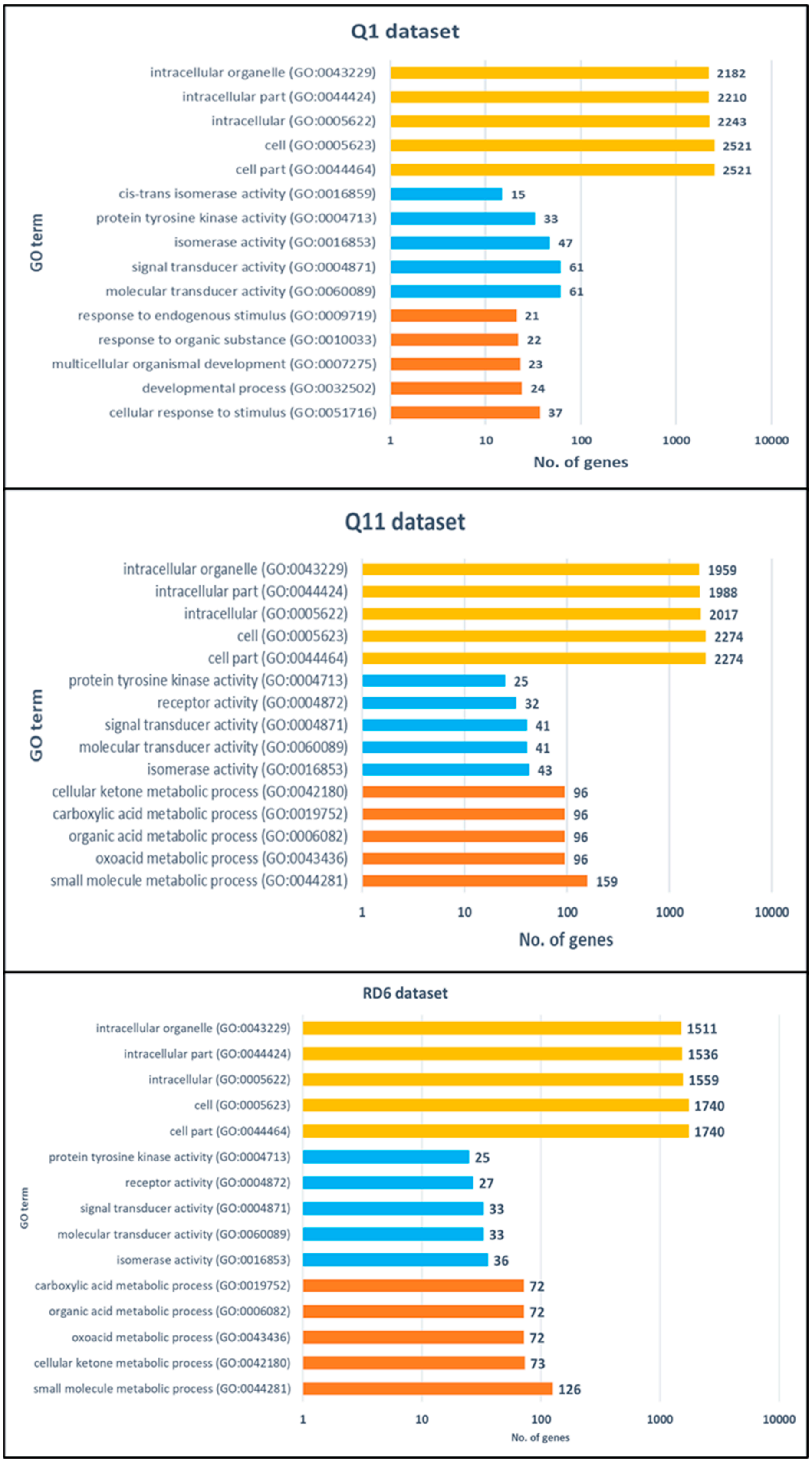

Figure S2. gene ontology analysis indicated the top five GO terms of down-regulated genes in Q1, Q11 and RD6 group, based on the number of genes in each GO term. Note: Biological process (brown bar), Molecular function (blue bar) and Cellular component (yellow bar)

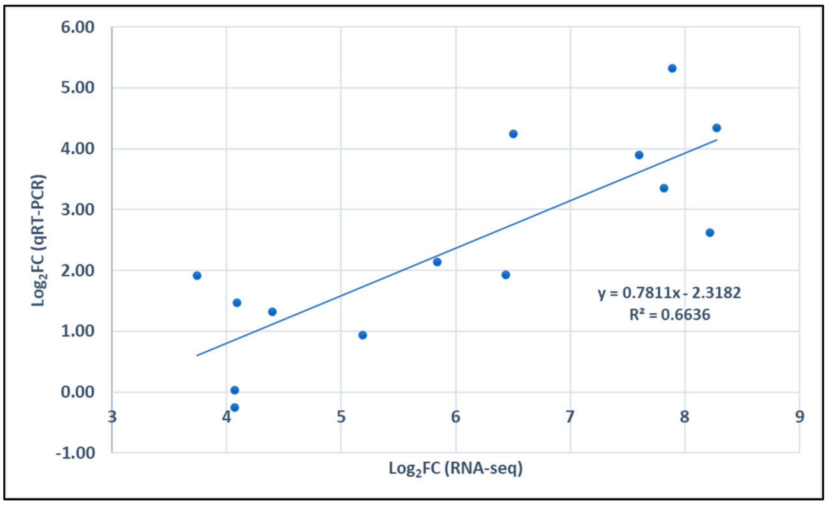

Figure S3. linear regression analysis indicated a correlation between the $\log_2$(Fold change) of RNA-seq and qRT-PCR.

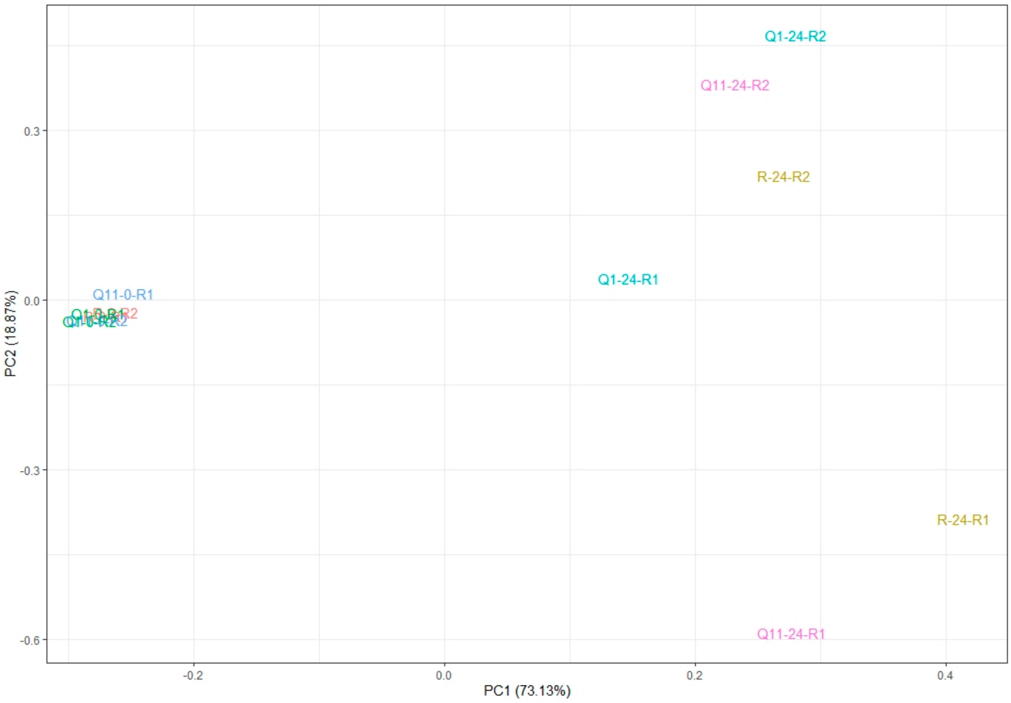

Figure S4. PCA plot of twelve RNA-seq data according to gene expression (FPKM)level.
